# From Senses to Memory During Childhood: A Systematic Review and Bayesian Meta-Analysis Exploring Multisensory Processing and Working Memory Development

**DOI:** 10.3390/ejihpe15080157

**Published:** 2025-08-12

**Authors:** Areej A. Alhamdan, Hayley E. Pickering, Melanie J. Murphy, Sheila G. Crewther

**Affiliations:** 1 Department of Psychology, Imam Muhammad Ibn Saud Islamic University (IMSIU), Riyadh 11564, Saudi Arabia; 2 Department of Psychology, Counselling and Therapy, La Trobe University, Melbourne, VIC 3086, Australia; 3 Centre for Mental Health and Brain Sciences, Swinburne University of Technology, Melbourne, VIC 3122, Australia

**Keywords:** systematic review, Bayesian meta-analysis, multisensory processing, working memory, children

## Abstract

Multisensory processing has long been recognized to enhance perception, cognition, and actions in adults. However, there is currently limited understanding of how multisensory stimuli, in comparison to unisensory stimuli, contribute to the development of both motor and verbally assessed working memory (WM) in children. Thus, the current study aimed to systematically review and meta-analyze the associations between the multisensory processing of auditory and visual stimuli, and performance on simple and more complex WM tasks, in children from birth to 15 years old. We also aimed to determine whether there are differences in WM capacity for audiovisual compared to unisensory auditory or visual stimuli alone after receptive and spoken language develop. Following PRISMA guidelines, a systematic search of PsycINFO, MEDLINE, Embase, PubMed, CINAHL and Web of Science databases identified that 21 out of 3968 articles met the inclusion criteria for Bayesian meta-analysis and the AXIS risk of bias criteria. The results showed at least extreme/decisive evidence for associations between verbal and motor reaction times on multisensory tasks and a variety of visual and auditory WM tasks, with verbal multisensory stimuli contributing more to verbally assessed WM capacity than unisensory auditory or visual stimuli alone. Furthermore, a meta-regression confirmed that age significantly moderates the observed association between multisensory processing and both visual and auditory WM tasks, indicating that verbal- and motor-assessed multisensory processing contribute differentially to WM performance, and to different age-determined extents. These findings have important implications for school-based learning methods and other educational activities where the implementation of multisensory stimuli is likely to enhance outcomes.

## 1. Introduction

Successful adaptation to constantly changing environments requires individuals to be able to attend to and process information from multiple senses simultaneously (Alais et al., 2010; Todd, 1912; Treisman & Gelade, 1980). From the day of birth, all five sensory systems (vision, sound, touch, taste, and smell) contribute to the emergence of conscious perception. with touch, taste, and smell dominating during the first few weeks of life (Maitre et al., 2020; Muir & Field, 1979), and vision dominating from around 10–16 weeks as the cortical binocular vision system becomes functionally active (Braddick & Atkinson, 2011; McCulloch & Skarf, 1991; Richards, 1985). However, it is the close temporal occurrence of multisensory stimuli, particularly vision and sound, that together facilitate the creation of a child’s perceptual world and conceptualization of their unique environment (Bahrick & Lickliter, 2000; Dionne-Dostie et al., 2015; Kopp, 2014; Neil et al., 2006). In particular, it is the regular multisensory combination of visual information in the environment and the hearing of consistently spoken words (e.g., a child’s own name) that leads to the rapid and early (within a few weeks) acquisition of aspects of basic receptive language by young animals and human infants (Mason et al., 2019). However, to date, there is little understanding of the neural pathways and factors affecting the developmental trajectory and achievement of adultlike multisensory motor integration and speech in children (Chartier et al., 2018; Kocsis et al., 2023; R. Wang et al., 2023). Thus, this review aims to explore the current literature relating to this issue.

### 1.1. Multisensory Development

Multisensory processing is typically defined in terms of the ability to establish neural connections between the neural networks associated with processing of temporally close sensory stimuli, which eventually enables the facilitation of more efficient motor and cognitive behaviour in young animals and children (Bremner et al., 2012; Stein et al., 2010), and adults (Todd, 1912; Treisman & Gelade, 1980). Indeed, as alluded to above, multisensory facilitation of sensorimotor information processing emerges early in the first year of life (such as a child turning upon hearing their name), long before the child gains adequate motor control to sit, walk, or speak (Mason et al., 2019). Neil et al. (2006) also observed multisensory facilitation of head and eye movements in response to spatially localized concurrent audiovisual stimuli in 2-month-old infants, and both Bahrick and Lickliter (2000); Kopp (2014) have reported enhanced responses to multisensory stimuli compared to unisensory stimuli in infants as young as six months old. Interestingly, it takes children another 18-24 months of physical and brain development, particularly biological development of the sensory, cognitive, and motor systems, to achieve the requisite basic speech and expressive language to adequately assess overall cognitive capacity, highlighting the complementary roles integration of motor control and sensory co-ordination play in multisensory processing and cognitive development (Chartier et al., 2018; Dionne-Dostie et al., 2015; D. J. Lewkowicz & Bremner, 2020; Soto-Faraco et al., 2012) especially verbally assessable working memory.

Multisensory development is also known to continue to facilitate faster and more precise visuomotor responses, even after formal schooling begins (Alhamdan et al., 2022; Barutchu et al., 2009; Brandwein et al., 2011; Nardini et al., 2016, 2008). Such enhanced multisensory motor reaction times have also been shown to be closely related to development of higher-order cognitive functions, such as memory and fluid intelligence (Barutchu et al., 2011; Denervaud et al., 2020b), and presumably also to the progressive development of motor control of speech pathways and verbal expressive language in young children (Alhamdan et al., 2023a). However, cognitive and neuropsychology research has seldom considered the early development and sophistication of the motor system required before either motor reaction times or verbal responses to multisensory stimuli can be measured. Thus, there remains a notable gap in the literature regarding understanding of how multisensory processing contributes to the development of motor reaction times (MRTs), speech multisensory processing, and working memory (WM) in children. It is also important to note that speech is a highly complex series of motor functions requiring multiple brain networks and areas (Guenther & Hickok, 2016; Kent, 2000; Maegherman et al., 2019; Simonyan & Horwitz, 2011), and potentially a limiting factor influencing the interpretation of the age at which various multisensory and cognitive tasks can be assessed. In fact, cognitive psychology has usually conceived of verbal speech as simple and largely automatic (Baddeley & Warrington, 1973), and hence not likely to require or impede the normally considered limited neural resources or receptive and expressive vocabulary available to individuals who differ with age. Therefore, this review aimed to systematically investigate the existing literature to better understand how concurrent audiovisual stimuli development contribute to both motor reaction times and verbal speech effect on the measurement of multisensory integration and verbally measured WM abilities across childhood.

### 1.2. Working Memory and Multisesnory Processing

Memory is an essential function of human cognition and daily life (Squire, 1992). As one of multiple memory networks, WM is traditionally defined as the ‘temporary maintenance and manipulation of a limited number’ of recently acquired (short-term memory; STM) or older long-term memory (LTM) information (Baddeley, 2003a, 2003b; Hitch & Baddeley, 1976) for a greater understanding of stimuli and their contribution to more complex semantic thinking (Buschman & Miller, 2022; Rossi et al., 2023; Weisberg & Reeves, 2013).

To date, cognitive research has almost exclusively focused on the role of auditory and verbal information in assessing development of WM (Baddeley, 2003a; Baddeley et al., 1998; Gathercole et al., 1992; Verhagen et al., 2019). Substantially less consideration has been given to development of visually based WM (H. E. Pickering et al., 2022), despite the known dominance of vision in driving attention and cognition, both subcortically and cortically in animals (Wiesel & Hubel, 1963), as well as the social interactions and language development of preverbal children (Moeskops et al., 2015; Padilla et al., 2015; Tzarouchi et al., 2009) and adult humans (Crewther et al., 1999; Goodale & Milner, 1992; Goldberg & Wurtz, 1972; Zackon et al., 1997). Indeed, the wealth of objective research available from primate electrophysiology post-1960s (Foxe & Simpson, 2005; Hubel & Wiesel, 1963; Goldberg & Wurtz, 1972), adult human psychophysics (Braddick & Atkinson, 2011; Crewther et al., 1999; Goodale & Milner, 1992), and brain imaging post 1990 (Corbetta et al., 1995) consistently demonstrate the dominance of visually driven attention and processing of more complex WM information, whether visually or auditorily derived via the parieto-frontal network (Corbetta & Shulman, 2002; D’Esposito, 2007).

Neuroimaging studies in adults have also consistently demonstrated the concurrent activation of the dorsolateral pre-frontal cortex via the parieto-frontal network and the neocerebellum during multisensory cognitive and sensory–motor tasks (Beauchamp et al., 2004; D’Esposito, 2007; Postle, 2006; D. Wang et al., 2025). Furthermore, in one of the few functional magnetic resonance imaging (fMRI) studies in children aged 8–13 years, Denervaud et al. (2020a) demonstrated that similar areas of blood-oxygen -level-dependent (BOLD) activation and functional connectivity associated with the visuo-parietal-frontal networks (Denervaud et al., 2020a). These findings align with the neural efficiency hypothesis, which proposes that multisensory input allows for more efficient cognitive processing by reducing redundancy and facilitating faster encoding and retrieval in adults (Haier et al., 1988; Stein & Stanford, 2008; Grady, 2012). Additionally, attentional control (Eysenck et al., 2007; Hopfinger & Slotnick, 2020) is enhanced when stimuli are multimodal (auditory and visual), as such stimuli are more effective in engaging and sustaining attention compared to unimodal stimuli (Macaluso & Driver, 2005; Shams & Seitz, 2008). Together, these findings suggest that multisensory integration not only affects relevant neural systems associated with WM but also provides cognitive advantages through binding, attention regulation, and WM load reduction during development (Denervaud et al., 2020b; Murray et al., 2016; Shimi et al., 2014).

As such, further investigation and clarification of the contributions from multisensory processing to auditory and visual attention and WM abilities should provide a more complete picture of the development of both domains in early childhood.

Substantial variability also exists in the literature regarding previously utilized paradigms of assessment of multisensory processing, and this may also reflect unconscious investigator prediction and perception of a child group’s maturity while impacting understanding of the developmental progression of the underlying skills. For example, Tremblay et al. (2007) have concluded that both verbally based multisensory processes as assessed with the McGurk Effect and nonverbal (flash–beep) multisensory motor responses follow different developmental time courses. This difference is likely associated with the ubiquitous observation that simple non-verbal motor multisensory processing reaches adult rates in early childhood (5–9-year-olds), whereas the complex neural motor pathways associated with both speech production (Chartier et al., 2018) and the cognitive development of language and extent of vocabulary continue throughout life (e.g., comparison of two older groups of 10–14 and 15–19 years; (Tremblay et al., 2007). Thus, the current review aims to incorporate the importance of considering the diversity into assessment paradigms when interpreting findings related to multisensory processing and WM development.

### 1.3. The Current Review

This review and meta-analysis aimed to systematically investigate current literature examining the association between multisensory processing and WM performance across childhood, with a focus on the type of multisensory tasks (i.e., manual motor vs. verbally assessed spoken multisensory tasks) and the ages of the participants. Previously published reviews of the existing multisensory literature in adults (Matusz et al., 2017; Quak et al., 2015) have consistently shown that multisensory presentation of information is generally associated with age improvement in accuracy in WM tasks, making meta-analysis necessary to determine these associations in schoolchildren (birth to 15-years). In addition, there is also considerable heterogeneity in the literature in terms of findings regarding the contribution of multisensory processing to WM capacity tasks in school age-children (Constantinidou & Evripidou, 2012; Gillam et al., 1998).

Therefore, the primary aims of the current review were as follows:

(i) We aimed to examine the association between motor reaction times (MRTs) and verbal multisensory processing for both visual and auditory WM measures. On the basis of past findings, studies were grouped according to the type of multisensory task and parameter being assessed (i.e., MRTs, or verbal tasks thus we hypothesized that accurate responses on verbal multisensory tasks (Constantinidou et al., 2011), and faster MRTs (Alhamdan et al., 2023b) would be significantly associated with better visual and auditory WM performance of children old enough to speak.
(ii) We sought to compare the contribution of unisensory (auditory-alone and/or visual-alone) to multisensory (audiovisual) stimuli to WM capacity across childhood. It was hypothesized, in line with past research in adults (Matusz et al., 2017; Tovar et al., 2020), that WM capacity (i.e., number of items able to be recalled) would be higher for audiovisual stimuli (i.e., cross-modal objects) than for unisensory stimuli (visual or/and auditory alone).

## 2. Method

### 2.1. Protocol and Registration

This review was conducted in accordance with the recommended protocols of the Preferred Reporting Items for Systematic Reviews and Meta-Analyses (PRISMA) guideline (Page et al., 2021) (see Supplementary Table S1 for PRISMA Checklist). Our protocol was pre-specified and registered in the International Prospective Register of Systematic Reviews (PROSPERO) under the existing registration number CRD42020148110 (Alhamdan et al., 2019), which is accessible https://www.crd.york.ac.uk/PROSPERO/view/CRD42020148110 (accessed on 29 July 2025). A summary of amendments to the protocol can be found in Supplementary Table S2.

### 2.2. Literature Search Strategy

The tailored search strategy was developed in collaboration with an academic librarian (HB) and executed in six electronic databases: PsycINFO (Ovid, from 1806), MEDLINE (Ovid, from 1946), EMBASE (Ovid, from 1947), PubMed, CINAHL (EBSCO), Web of Science (ISI) using MeSH terms as appropriate, as well as Google Scholar, for all available years. We also incorporated additional terms suggested by certain databases when they were deemed relevant (e.g., intersensory processing). The initial search in the database was conducted on the 29th and 30th of July 2019, with an updated the search using the same protocol conducted on the 11th of August 2023 to include newly published articles. The detailed search strategy employed for the MEDLINE (Ovid) database is presented in Table 1. The specific search strategy for all databases is presented in Supplementary Table S3, while Supplementary Figures S1–S6 provide the specific search strategies for each database.

### 2.3. Eligibility Criteria and Study Selection

To be eligible for inclusion, studies were required to meet the following criteria. Firstly, studies were required to be quantitative, peer-reviewed, and written in English, with no limitations on the publication year. Qualitative papers, case studies, training programmes, reviews, book chapters, and dissertations were excluded. Studies of children with a neurodevelopmental disorder (e.g., ADHD, ASD, Language Disorder) or hearing or visual impairment that did not include neurotypical children as controls were also excluded. Furthermore, studies were only included if they employed correlation analyses comparing a multisensory processing task and a measure of visual or auditory WM, or if they assessed WM capacity (i.e., number of items able to be recalled) using three different types of stimuli (audiovisual, auditory, and visual stimuli) within the same task to allow for effect size comparisons between conditions.

All studies retrieved from the databases matching the search strategy were imported into Covidence, an online software platform designed for high-quality systematic reviews and to assist researchers in efficiently tracking each document through the inclusion/exclusion criteria process (Veritas Health Innovation, 2019). For the initial search, two reviewers (AA and HP) independently screened all titles and abstracts from each identified record to decide whether to exclude the study or include it for further review. Subsequently, both reviewers (AA and HP) reviewed the method and results sections of each potentially relevant study to make a final decision regarding the inclusion and exclusion criteria. These steps were completed by one reviewer (AA) for the second search. In cases of disagreement between the two reviewers, consultation with a co-author (SC) was sought for resolution.

The decision to exclude a study was based on a custom hierarchy consisting of seven exclusion criteria, as outlined below:

1. Studies not published in English;
2. Study design that did not meet criteria (e.g., case studies, reviews, qualitative papers, training programs, book chapters or dissertations);
3. Sample/s that did not meet the inclusion criteria (e.g., participants over 15 years old and/or individuals with neurodevelopmental disorders, hearing impairments, or visual impairments);
4. No measure of multisensory processing (i.e., audiovisual stimuli);
5. No measure of WM;
6. Required data/statistics not reported (e.g., no correlation, or the standard deviation for each group could not be extracted from the provided data);
7. Study did not report the result on typically developing children separately from atypically developing children.

Where the full text of a study was unavailable, corresponding authors were contacted via email. Possible related articles were excluded if the full text could not be obtained, if there was insufficient information to determine if the study met the inclusion criteria, or if communication with the corresponding author proved ineffective in obtaining the required information.

### 2.4. Risk of Bias and Quality Assessment

The methodological quality of the included studies was evaluated using the AXIS tool (Appraisal Tool for Cross-Sectional Studies; Downes et al., 2016). The 20 items included in this appraisal are intended to achieve the following objectives: clarify the study aims and objectives, evaluate the appropriateness of the study design and methodology, assess the validity of the results, ascertain whether the discussion and conclusion are well-supported by the results, and review ethical considerations. To facilitate the comparison of study quality, we followed the procedures used by H. E. Pickering et al. (2022) by transforming outcomes into percentages, where a higher percentage indicated better study quality. These percentages were calculated based on the number of relevant items for each specific study, excluding items marked as ‘Not Applicable’. Studies achieving a rating of 75% or higher were categorized as high-quality, those with scores ranging from 50% to 74% were considered moderate quality, and studies with scores below 50% were categorized as poor quality. Only studies with a quality score of 50% or higher were included in the review. Table 2 presents the quality qssessment and summary of critical appraisal of the included studies using (AXIS) tool.

### 2.5. Data Extraction

The following information from each study was extracted: study details (author(s), year of publication), study purpose (aims as stated in the study), participant information (number of total participants, age [M, (SD), range]), measures of multisensory processing tasks (type of MS task, task description, and degree of complexity), measures of WM tasks (visual or auditory), and outcomes for both correlation coefficients and effect size analyses (see Table 3 and Table 4).

The first outcome extracted was the bivariate correlation *r* between a multisensory measure and WM task. We acknowledge that “*r*” is typically reported in experimental designs; however, in some studies where correlation is not the primary interest, it may not be included (Dunlap et al., 1996). Therefore, “*r*” may not always be available unless the raw data can be obtained. To address this issue Borenstein (2009) recommend estimating the correlation by referring to related studies and conducting sensitivity analyses using a range of reasonable correlation values. Another approach suggested by Hullett and Levine (2003) involves estimating “*r*” values using available *t* and *f* statistics. However, when these specific statistics were not accessible, effect size was directly estimated using means and standard deviations (Dunlap et al., 1996; Morris & DeShon, 2002). In the current review, effect sizes using Cohen’s *d* were computed for the comparison of WM capacity to audiovisual stimuli vs. auditory stimuli in the first analysis, and for WM capacity to audiovisual stimuli vs. visual stimuli in the second analysis.

To synthesize the correlation data, we categorized multisensory tasks into three groups:

1. Motor reaction times (i.e., measuring how quickly participants press a button);
2. Verbal multisensory tasks (i.e., assessing accuracy through the number of correct responses as reported verbally and without any time related data);
3. Motor non-timed tasks (i.e., tasks involving manual motor actions but focusing on the accuracy of responses rather than response time).

Two studies (Broadbent et al., 2018; Cleary et al., 2001) and two datapoints from Barutchu et al. (2020) did not fit this categorization of multisensory tasks, as they involved motor but non-timed tasks. Hence, they have been discussed in the narrative review, and their results are reported in Table 5.

For group difference (effect size) data synthesis, we categorized multisensory WM tasks based on two comparisons: (i) the contribution of audiovisual stimuli (AVS) vs. auditory stimuli (AS) to WM capacity, and (ii) the contribution of audiovisual stimuli (AVS) vs. visual stimuli (VS) to WM capacity. Complete data extractions, including means, N, and SD for each group to calculate effect sizes for group difference studies, are presented in Supplementary Table S4. In cases of unclear or missing information within the publications, we attempted to contact the authors for further clarification or additional data.

### 2.6. Data Analysis

Studies were grouped into two different analyses. First, correlation coefficients were used to investigate the association between multisensory tasks (including motor and verbal multisensory tasks) and WM. Fisher transformations, *r*-to-*Z* transformations, were employed to convert the correlation coefficients into effect size-based Fisher’s *Z* (Silver & Dunlap, 1987). Standard error was computed based on the sample size of each study. In some cases, studies presented multiple correlations of interest for a single sample. Effect size was categorized as weak (ranging from 0.1 to 0.3), moderate (ranging from 0.4 to 0.6), and strong (ranging from 0.7 to 0.9), following the classification proposed by (Akoglu, 2018).

Secondly, effect sizes were computed to examine the differences in WM capacity for audiovisual stimuli vs. auditory stimuli, as well as for audiovisual stimuli vs. visual stimuli (VS). Cohen’s *d* was computed using the standard deviation (SD) and sample size (n) for each group, allowing for the estimation of effect size across studies, with 95% credible intervals (CI). Cohen’s *d* was chosen as a robust measure of effect sizes for sample sizes larger than 20 (Borenstein et al., 2021). Multiple effect sizes for a single sample were extracted in some studies. Effect sizes of 0.30, 0.50, and 0.80 represented small, medium, and large effects, respectively (Cohen, 1988).

Bayesian meta-analyses were conducted using the free software JASP; Jeffreys’s Amazing Statistics Program 0.16.3.0 (JASP Team, 2022; http://www.jasp-stats.org/ (accessed on 30 Aguest 2022)). A random effect model is most appropriate for current data rather than fixed effect as it is well-suited for situations where sources beyond sampling error may contribute to the heterogeneity observed in effect sizes between studies (Langan et al., 2019; Thompson & Higgins, 2002). Bayesian analyses were selected to help in addressing the issue of variability underestimation, providing a more flexible and robust framework and straightforward interpretation (Hackenberger, 2020; Marsman & Wagenmakers, 2017). In addition, the Bayesian approach assists researchers to address publication bias and offers the advantage of sequential analysis, (Gronau et al., 2021), and this approach offers theoretical and practical advantages in the evaluation and interpretation of developmental data (Weaver & Hamada, 2016). For the effect size (μ), we used the default Cauchy prior distribution with scale of 1/$\sqrt{2}$ ≈ 0.707, and the default Inverse-Gamma [1, 0.15] as the prior distribution on heterogeneity (τ) (Gronau et al., 2021). The prior (estimated) and posterior (observed) odds and 95% credible intervals (95% CI) are reported. Bayesian Factors (BF) are also reported, where higher BF_10_ values are interpreted as strength of evidence in favour of the alternative hypothesis (H_1_) against the null hypothesis (H_0_) testing. Heterogeneity (BF_rf;_ variance between studies) was conducted to assess the presence or absence of between-study variability, with Bayes Factor estimates providing evidence for or against the null hypothesis of homogeneity.

## 3. Results

### 3.1. Study Selection

**Figure 1:**
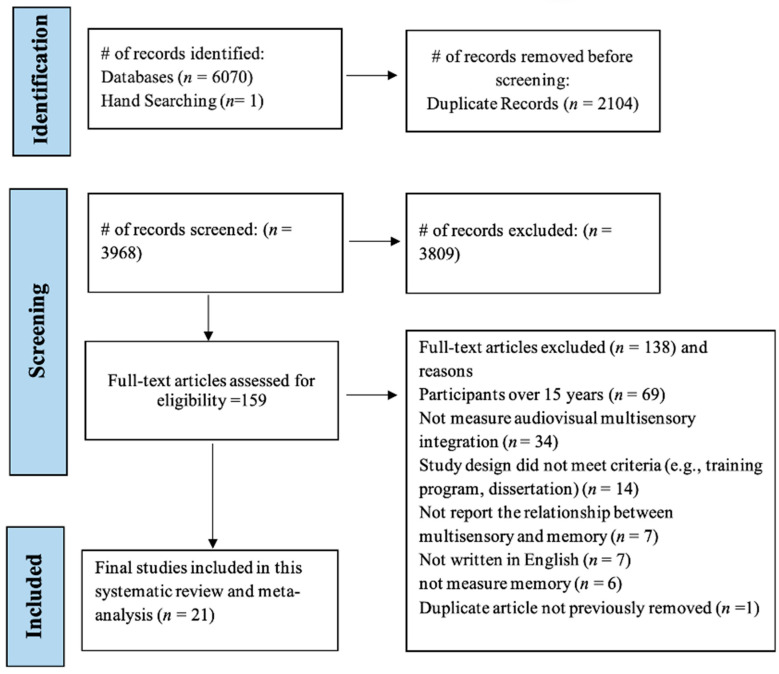
PRISMA flow diagram of study selection process for review.

The database searches yielded 6070 records, of which 3968 were unique records (see Supplementary Material Figures S1–S6 for a detailed breakdown of records per database), and 61 studies were found via other sources (i.e., manual searching and Google Scholar). After individually assessing the titles and abstracts, 159 records met the criteria for full-text review. Of these, 138 articles were excluded during the full-text review, and one further duplicated article was detected. Thus, 21 studies were ultimately included in the systematic review and meta-analysis. The study selection process is illustrated in the PRISMA flow diagram in Figure 1.

### 3.2. Quality Assessment and Risk of Bias

The AXIS tool (Downes et al., 2016) was employed to assess the quality of the 21 selected studies. All studies met the required quality standards and were therefore included in the review and meta-analyses. Six studies achieved a moderate quality rating with an average percentage of 69% (60–73%), whereas fifteen studies demonstrated high quality with an average percentage of 83% (76–94%).

All studies included in the review had clear aims/objectives, employed appropriate study designs to meet their stated aims, and presented well-justified conclusions. However, only one of the 21 studies included statistical justification of sample size (Alhamdan et al., 2023b). Ethical approval and the use of consent forms were not described in eight studies (Badian, 1977; Cleary et al., 2001; Crawford & Fry, 1979; Field & Anderson, 1985; Gillam et al., 1998; Hatchette & Evans, 1983; Magimairaj et al., 2009; Montgomery et al., 2009). Seven studies also failed to include a discussion of limitations to their methodological approach (Badian, 1977; Barutchu et al., 2011; Cleary et al., 2001; Constantinidou et al., 2011; Crawford & Fry, 1979; Hatchette & Evans, 1983; Pillai & Yathiraj, 2017b).

In summary, the majority of the studies that were included in this review provided sufficient information and demonstrated a high level of quality. All 21 studies employed appropriate methodologies for data collection and featured samples that effectively represented the children they targeted. Consequently, all 21 studies were included in this systematic review and meta-analysis. A summary of the quality assessment is presented in Table 2.

**Table 2:** Summary of critical appraisal of the included studies using (AXIS) tool.

Study Details			Intro	Method										Results					Discussion		Other		Total
#	(Author(s), Year)	Study Design Contributed to This Meta-Analysis	1: Aims	2: Study Design	3: Sample Size Justification	4: Target Population	5: Sampling Frame	6: Sample Selection	7: Non-Responders	8: Appropriate Measurement	9: Reliable Measurement	10: Statistical Significance	11: Repeatability	12: Basic Data	13: Response Rate	14: Non-Responders	15: internal Consistency	16: All Results Presented	17: Conclusions Justified	18: Limitations Discussed	19: Funding a& COI Declared	20: Ethics & Consent	%
1	(Alhamdan et al., 2023b)	Correlation	●	●	●	●	●	●	●	●	●	●	●	●	●	●	●	●	●	●	●	●	94%
2	(Badian, 1977)	Correlation	●	●	●	●	●	●	●	●	●	●	●	●	●	●	●	●	●	●	●	●	69%
3	(Barutchu et al., 2011)	Correlation	●	●	●	●	●	●	●	●	●	●	●	●	●	●	●	●	●	●	●	●	82%
4	(Barutchu et al., 2019)	Correlation	●	●	●	●	●	●	●	●	●	●	●	●	●	●	●	●	●	●	●	●	87%
5	(Barutchu et al., 2020)	Correlation	●	●	●	●	●	●	●	●	●	●	●	●	●	●	●	●	●	●	●	●	84%
6	(Broadbent et al., 2018)	Correlation	●	●	●	●	●	●	●	●	●	●	●	●	●	●	●	●	●	●	●	●	79%
7	(Choudhury et al., 2007)	Group Differences	●	●	●	●	●	●	●	●	●	●	●	●	●	●	●	●	●	●	●	●	85%
8	(Cleary et al., 2001)	Correlation	●	●	●	●	●	●	●	●	●	●	●	●	●	●	●	●	●	●	●	●	70%
9	(Constantinidou et al., 2011)	Both	●	●	●	●	●	●	●	●	●	●	●	●	●	●	●	●	●	●	●	●	78%
10	(Constantinidou & Evripidou, 2012)	Group Differences	●	●	●	●	●	●	●	●	●	●	●	●	●	●	●	●	●	●	●	●	84%
11	(Crawford & Fry, 1979)	Correlation	●	●	●	●	●	●	●	●	●	●	●	●	●	●	●	●	●	●	●	●	60%
12	(Davies et al., 2019)	Correlation	●	●	●	●	●	●	●	●	●	●	●	●	●	●	●	●	●	●	●	●	80%
13	(Denervaud et al., 2020b)	Correlation	●	●	●	●	●	●	●	●	●	●	●	●	●	●	●	●	●	●	●	●	84%
14	(Field & Anderson, 1985)	Group Differences	●	●	●	●	●	●	●	●	●	●	●	●	●	●	●	●	●	●	●	●	76%
15	(Gillam et al., 1998)	Group Differences	●	●	●	●	●	●	●	●	●	●	●	●	●	●	●	●	●	●	●	●	81%
16	(Hatchette & Evans, 1983)	Group Differences	●	●	●	●	●	●	●	●	●	●	●	●	●	●	●	●	●	●	●	●	73%
17	(Montgomery et al., 2009)	Correlation	●	●	●	●	●	●	●	●	●	●	●	●	●	●	●	●	●	●	●	●	76%
18	(Magimairaj et al., 2009)	Correlation	●	●	●	●	●	●	●	●	●	●	●	●	●	●	●	●	●	●	●	●	84%
19	(Ortiz-Mantilla et al., 2008)	Group Differences	●	●	●	●	●	●	●	●	●	●	●	●	●	●	●	●	●	●	●	●	89%
20	(Pillai & Yathiraj, 2017a)	Correlation	●	●	●	●	●	●	●	●	●	●	●	●	●	●	●	●	●	●	●	●	79%
21	(Pillai & Yathiraj, 2017b)	Group Differences	●	●	●	●	●	●	●	●	●	●	●	●	●	●	●	●	●	●	●	●	72%

Note. Green = yes; red = no; yellow = unclear; blue = not applicable; intro = introduction; COI = conflict of interest. Rating of “75% or higher” = high quality; “50% to 74%” = moderate quality; “50% and below” = poor quality (H. E. Pickering et al., 2022). AXIS appraisal tool for cross-sectional studies.

### 3.3. Study Characteristics and Data Synthesis

**Figure 2:**
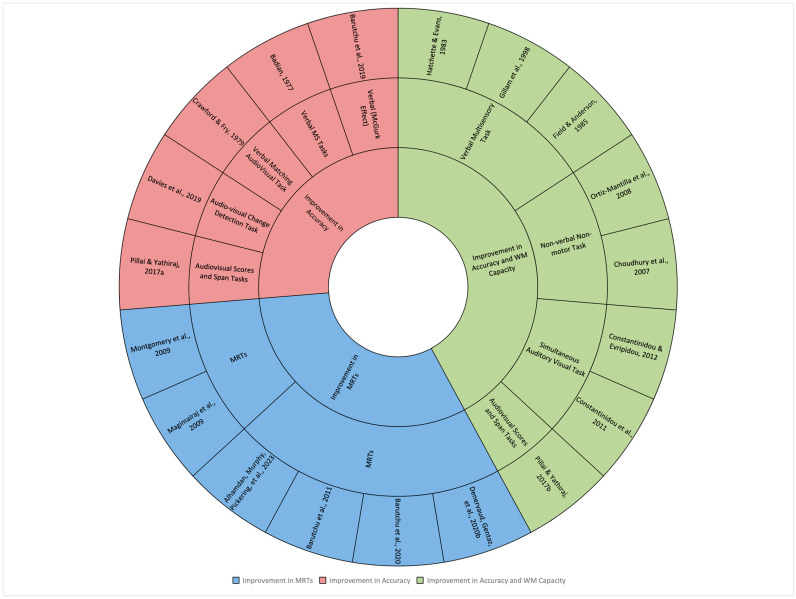
A starburst visualization representing multisensory processing improvements, assessment tasks used to assess skills, and study sources identified following systematic review and Bayesian meta-analysis. This figure presents a hierarchical visualization of findings from a Bayesian meta-analysis examining visual, auditory, audiovisual multisensory, and working memory abilities in childhood. The inner ring represents the types of multisensory task where improvements were observed. The middle ring depicts the specific tasks used to assess these multisensory processing. The outer ring reports the study authors reporting the observed patterns. This layered representation facilitates an integrated understanding of the relationships between multisensory processing skills and WM performance, their operationalisation, and the supporting evidence base. (Source studies: Alhamdan et al., 2023b; Barutchu et al., 2011, 2019, 2020; Badian, 1977; Crawford & Fry, 1979; Choudhury et al., 2007; Constantinidou & Evripidou, 2012; Constantinidou et al., 2011; Davies et al., 2019; Denervaud et al., 2020b; Field & Anderson, 1985; Gillam et al., 1998; Hatchette & Evans, 1983; Magimairaj et al., 2009; Montgomery et al., 2009; Ortiz-Mantilla et al., 2008; Pillai & Yathiraj, 2017a, 2017b).

Overall, 19 of the 21 included studies contributed to the correlation and group differences meta-analyses. Specifically, 11 studies (58%) provided correlation data, which was categorized into two meta-analyses (based on MRTs and verbal multisensory tasks, see Table 3; for description of tasks). Additionally, 7 studies (36%) provided data for estimating effect sizes (to compare WM capacity for audiovisual stimuli versus unisensory stimuli). One study (6%) included both types of data (correlation and group differences) (Table 4). The remaining two studies (Broadbent et al., 2018; Cleary et al., 2001) and two datapoints from Barutchu et al. (2020) involved non-timed motor tasks and hence could not be included in the meta-analyses, providing six datapoints in a narrative synthesis presented in Table 5. A starburst visualization summarizing the various types of multisensory tasks, their corresponding assessment tools, and the contributing study sources is shown in Figure 2.

**Table 3:** Description of multisensory tasks in all included studies.

#	(Author(s), Year)	Type of MS Task	Task Description	Aim of MS Task
1	(Alhamdan et al., 2023b)	MRTs Non-verbal motor task (Simple AV Task)	Multisensory processing was measured using motor reaction times to target detection. The targets included three types of stimuli: an auditory stimulus (AS; beep), a visual stimulus (VS; grey circle), and an audiovisual stimulus (AVS; beep and grey circle presented simultaneously) The children aged 5-10 years were instructed to press a button as rapidly as possible	Multisensory gain seen as measure of improvement in MRT (the time it took participants to respond in milliseconds)
2	(Barutchu et al., 2011)	MRTs Non-verbal motor task (Simple AV Task)	Participants aged 7–11 years were randomly presented with AS, VS, AVS, and blank stimuli and instructed to press a button when they saw a flash, when they heard a tone burst, or when both happened simultaneously	Multisensory gain seen as measure of improvement in MRT (the time it took participants to respond in milliseconds)
3	(Barutchu et al., 2020)	MRTs Non-verbal motor task (Simple AV Task and Associate Learning Taks; ALT)	Simple detection task is similar to the task used by the same authors 2011, while ALTs required participants aged 8–11 years to learn an association between novel black symbols and either a novel auditory sound (novel-AV), or a verbal sound (verbal-AV) with a consonant-vowel-consonant	Multisensory gain seen as measure of improvement in MRT (the time it took participants to respond in milliseconds)
4	(Denervaud et al., 2020b)	MRTs Non-verbal motor task (Simple AV Task)	Participants aged 4–15 years were randomly presented with AS, VS, AVS, and blank stimuli and instructed to press a button when they saw a flash, when they heard a tone burst, or when both happened simultaneously	Multisensory gain seen as measure of improvement in MRT (the time it took participants to respond in milliseconds)
5 & 6	(Montgomery et al., 2009) & (Magimairaj et al., 2009)	MRTs Non-verbal motor task (Colour–word Recognition)	Participant aged 6–12 were asked to touch a coloured shape (e.g., a green square) as quickly as possible in response to hearing a familiar colour word (e.g., “green”). The task required them to make a simple, speeded decision about which visual object matched the spoken color name.	Multisensory gain seen as measure of improvement in MRT (the time it took participants to respond in milliseconds)
7	(Badian, 1977)	Verbal MS Tasks	In the verbal MS task, participants aged 8–11 years were presented with grouped sequences of the names of four geometric forms: square, circle, triangle, and cross. The auditory stimuli involved orally presenting these names, while the corresponding visual stimuli consisted of sequences of the geometric forms drawn in black outline on white cards. Participants were required to match or select the correct visual response based on the auditory input	Multisensory gain seen as measure of improvement in accuracy (the proportion of correct responses on trials)
8	(Barutchu et al., 2019)	Verbal (McGurk Effect)	Participants aged 8–11 years were shown incongruous auditory syllables and visual syllables in four different videos (i.e., three matching videos and one incongruent audiovisual video with /ba-ba/ sounds and /ga-ga/ lip movements)	Multisensory gain seen as the number of perceptions of the fused percept (e.g., “da” or “tha”)
9	(Broadbent et al., 2018)	Motor non-timed Task (Multisensory Attention Learning Task (MALT)	The Multisensory Attention Learning Task (MALT) studies how sensory cues affect attention and learning. Participants aged 6–10 years view frog images, for example, paired with frog sounds in three conditions, visual, auditory, and combined cues for frog categorization. They respond to target frogs while disregarding others. Participants are instructed to press the space bar whenever a frog (the target animal) appears on the screen	Improvement in accuracy
10	(Cleary et al., 2001)	Motor non-timed Task (Auditory-plus-visual–spatial memory game)	Participants aged 8–9 years were instructed to press appropriate buttons on the response box when hearing sounds through the loudspeaker and seeing the buttons light up. For example, if they heard ‘blue’ followed by ‘green’ (indicating two buttons consecutively), they were expected to press the blue button first and then the green button	Improvement in accuracy
11 & 12	(Constantinidou et al., 2011) & (Constantinidou & Evripidou, 2012)	Verbal Auditory, Visual, and Simultaneous Auditory visual Task	Participants aged 7–13 years were presented words and pictures under three conditions: (a) auditory, (b) visual, and (c) auditory plus visual, and they asked to recall the items after each presentation	Multisensory gain seen as measure of improvement in accuracy (the proportion of correct responses on trials)
13	(Crawford & Fry, 1979)	Verbal (Matching Auditory and Visual trigrams)	Participants aged 5–9 years completed four matching tasks: two within the same senses (visual-to-visual, auditory-to-auditory) and two between senses (visual-to-auditory, auditory-to-visual). They matched cards with sounds and images, following instructions based on names given through cards or a tape recorder	Multisensory gain seen as measure of improvement in accuracy (the proportion of correct responses on trials)
14	(Davies et al., 2019)	Audiovisual Change Detection Task	Participants aged 5–6 years were presented with two sequential memory displays, each of which contained one shape and one sound. They were asked if the test shape-sound pairing was the same or different to either of the original pairings in the memory displays	Multisensory gain seen as measure of improvement in accuracy (the proportion of correct responses on trials)
15 & 16	(Choudhury et al., 2007) & (Ortiz-Mantilla et al., 2008)	Habituation Recognition Memory Taks Non-verbal non-motor task	Infants (6–9 months) participated in a series of habituation-recognition memory tasks, including one task involving visual stimuli with familiarized and novel faces, and two tasks combining auditory and visual stimuli. These tasks featured abstract visual slides paired with tone-pairs (with durations of 70 ms and 300 ms, as described in Choudhury et al., 2007), as well as consonant-vowel (CV) syllables, as reported in Ortiz-Mantilla et al., 2008. During the habituation phase, infants underwent consecutive discrete trials based on their individual looking times	Improvement in looking time
17	(Field & Anderson, 1985)	Cued and Free Recall Measures of Television Program Verbal multisensory task	Participants aged 5- and 9-years were presented with a 35 min colour videotape containing six short segments of story, including three types of modalities (visual, auditory and audiovisual content). Participants were then asked cued and free questions for each story	Improvement in accuracy and capacity of WM
18	(Gillam et al., 1998)	Audiovisual recall task-digits Verbal multisensory task	Participants aged 8–12 years were presented with (a) auditory digits spoken in a male voice, (b) visual digits appearing on a computer screen, and (c) audiovisual digits appearing on a computer screen in synchrony with the auditory presentation. They were asked to recall the digit in the same order as they appeared in the presentation	Improvement in accuracy and capacity of WM
19	(Hatchette & Evans, 1983)	Multisensory temporal/spatial Pattern Matching Taks Verbal multisensory task	Participants aged 7–10 years were presented with four to nine black dot pictures with either one or two discontinuities in patterns, and auditory stimuli. In the multisensory condition, the visual and audio stimuli were presented simultaneously, and participants were asked to determine whether the items presented were the same or different	Improvement in accuracy and capacity of WM
20 & 21	(Pillai & Yathiraj, 2017a) & (Pillai & Yathiraj, 2017b)	Visual and Audiovisual Scores and Span Tasks	Participants aged 7–9 years were instructed to repeat the words of the sequence presented auditory, visual and auditory–visual combined modalities and each memory skill was calculated using two different scoring procedures (score and span)	Improvement in accuracy and capacity of WM

Note. MRTs = motor reaction times; WM = working memory.

**Table 4:** Characteristics of studies included in meta-analyses.

Study Details				Participants		Measures		Meta-Analysis Outcomes
#	Citation	Aim as in Study	Study Design	*N*	Age M (SD); Range	Multisensory Task/s	Memory Task/s	Meta-Analysis Category Fishers’ z/Cohens’ *d* [95% CI]
1	(Alhamdan et al., 2023b)	To investigate developmental changes in the associations between age, and visual and auditory short-term and WM, and multisensory processing	Correlation	75	7.95 (1.70) 5–10 years	Simple Audiovisual Detection Task	“VWM and AWM” using (Digit Span Tasks)	1. AV−MRTs vs. VWM (Forwards) −0.40 [−0.63, −0.17] 2. AV−MRTs vs. VWM (Backwards) −0.55 [−0.78, −0.32] 3. AV−MRTs vs. AWM (Forwards) −0.38 [−0.65, −0.15] 4. AV−MRTs vs. AWM (Backwards) −0.45 [−0.68, −0.22]
2	(Badian, 1977)	To explore the nature of auditory–visual processing and memory performance	Correlation	30	9.71 (11.95) 8–11 years 0	Verbal Multisensory Tasks	“AWM” Auditory Memory Task (AMT) and the Auditory Sequential Memory subtest (ITPA)	1. MS (verbal) vs. AMT 0.21 [−0.16, 0.59] 2. MS (verbal) vs. ITPA 0.29 [−0.09, 0.66]
3	(Barutchu et al., 2011)	To investigate the relationships between multisensory integration, auditory background noise, and the general intellectual abilities and AWM	Correlation	85	9.7 7–11 years	Simple Audiovisual Detection Task	“AWM” WISC–IV WMI	AV−MRTs vs. AWM −0.13 [−0.35, 0.09]
4	(Barutchu et al., 2019)	To investigate developmental relationships between attention, multisensory processes, and children’s general intellectual abilities including AWM	Correlation	51	9.18 (1.61) 7–13 years	McGurk Effect/Illusion Task	“AWM” WISC–IV WMI	MS (McGurk) vs. AWM −0.13 [−0.41, 0.15]
5	(Barutchu et al., 2020)	To investigate the developmental profile and relationships between uni- and multisensory processes and children’s intellectual abilities including AWM	Correlation	41	9.85 8–11 years	Simple Audiovisual Detection Task Associative Learning Tasks (ALT)	“AWM” WISC–IV WMI	1. AV−MRTs vs. AWM 0.33 [0.00, 0.66] 2. AV−MRTs (ALT−Novel) vs. AWM 0.17 [−0.16, 0.50] 3. AV−MRTs (ALT−Verbal) vs. AWM −0.06 [−0.39, 0.27]
6	(Crawford & Fry, 1979)	To explore the contributions of auditory and visual WM to audiovisual multisensory stimuli	Correlation	52	6.75 5–9 years	Matching Auditory and Visual trigrams	“VWM” Visual-Sequential Memory subtest (ITPA), and “AWM” WISC (Digit Span)	1. Verbal (A−V) vs. Total−WM 0.55 [0.27, 0.83] 2. Verbal (V−A) vs. Total−WM 0.69 [0.41, 0.97] 3. (A−V) vs. VWM 0.52 [0.24, 0.80]
7	(Davies et al., 2019)	To test the developmental increase in audiovisual binding ability and its influence on retrieving bound audiovisual information and measures of verbal and visual complex WM span	Correlation	49	5.6 (3.25) 5–6 years	Audiovisual Change Detection Task	“AWM” Listening Recall Task and “VWM” Odd One Out Task	1. Verbal (A−V binding) vs. AWM 0.65 [0.36, 0.94] 2. Verbal (A−V binding) vs. VWM 0.60 [0.32, 0.89]
8	(Denervaud et al., 2020b)	To investigate the relationship between multisensory processing and higher-level cognition such as WM, and fluid intelligence in school children	Correlation	77	8.1 (3.0) 4–15 years	Simple Audiovisual Detection Task	“AWM” WISC (Digit Span)	AV−MRTs vs. AWM −0.56 [−0.79, −0.33]
9	(Montgomery et al., 2009)	To examine the potential influence of three main components of WM, and processing speed (AV reaction time) on children’s ability to understand spoken narrative	Correlation	67	8.39 (1.69) 6–11 years	Auditory–visual MRTs Task (colour word recognition)	“AWM” WISC (Digit Span), and Concurrent processing-storage (CPS)	1. AV−MRTs vs. AWM (DS) −0.46 [−0.70, −0.21] 2. AV−MRTs vs. AWM (CPS) −0.51 [−0.76, −0.27]
10	(Magimairaj et al., 2009)	To investigate the relative contribution of storage, processing speed (AV reaction time), and attentional allocation on digits and complex span tests	Correlation	65	8.6 (1.8) 6–12 years	Auditory–visual MRTs Task (colour word recognition)	“AWM” WISC (Digit Span), and Complex Memory Span Task (CMST)	1. AV−MRTs vs. AWM (CMST) −0.44 [−0.68, −0.19] 2. AV−MRTs vs. AWM −0.24 [−0.49, 0.00]
11	(Pillai & Yathiraj, 2017a)	To test whether there are associations between different memory skills and the auditory, visual, and combined modalities	Correlation	28	8.1 7–8 years	Auditory, Visual and Audiovisual Scores and Span Tasks	Revised Auditory Memory and Sequencing Test (RAMST-IE)	1. Verbal MS vs. AWM (RAMST−Span) 0.42 [0.03, 0.82] 2. Verbal MS vs. AWM (RAMST−Score) 0.89 [0.50, 1.28]
12	(Constantinidou et al., 2011)	To investigate the effects of stimulus presentation modality (i.e., auditory, visual and audiovisual) on WM performance	Correlation & Group differences	40	9.85 (1.03) 7–13 years	Auditory, Visual, and Simultaneous Auditory visual Task	“AWM” CVLT-C (Listening Recall Task)	Correlation Verbal (A−V) vs. AWM 0.38 [0.06, 0.69 Group Differences 1. WMC−AVS vs. WMC−AS (M= 8 years) 0.32 [−0.28, 0.91] 2. WMC−AVS vs. WMC−AS (M = 10 years) 0.75 [0.07, 1.42] 3. WMC−AVS vs. WMC−VS (M = 8 years) 0.07 [−0.52, 0.67] 4. WMC−AVS vs. WMC−VS (M = 10 years) −0.19 [−0.84, 0.47]
13	(Constantinidou & Evripidou, 2012)	To investigate the effects of stimulus presentation modality on WM performance in children	Group differences	20	11.5 (5.07) 10–12	Auditory, Visual, and Simultaneous Auditory visual Task using AVLT (Auditory Verbal Learning Test Paradigm)		1. WMC-AVS vs. WMC-AS 0.23 [−0.39, 0.86] 2. WMC-AVS vs. WMC-VS −0.28 [−0.91, 0.34]
14	(Choudhury et al., 2007)	To determine if performance on infant information processing measures differ as a function of family history of specific language impairment or the particular demand characteristics of two different Rapid Auditory Processing paradigms used	Group differences	29	8.97 mo (1.0) 6–9 mo	Auditory Visual Habituation Recognition Memory Task		1. AVH/RM (70 ms) vs. VH/RM 0.25 [−0.27, 0.76] 2. AVH/RM (300 ms) vs. VH/RM 0.12 [−0.39, 0.64]
15	(Ortiz-Mantilla et al., 2008)	To determine whether performance differences on language and cognitive tasks were due to poorer global cognitive performance or to deficits in specific processing abilities	Group differences	32	8.97 mo (0.58) 6 & 9 mo	Auditory Visual Habituation Recognition Memory Task		1. AVH/RM (6 mo) vs. VH/RM 0.20 [−0.29, 0.70] 2. AVH/RM (9 mo) vs. VH/RM −0.12 [−0.61, 0.37]
16	(Field & Anderson, 1985)	To investigate how children’s visual orientation and memory recall of a video clip are influenced by different factors such as the emphasis on different modalities (visual, auditory, or both)	Group differences	80	5.1 & 9.1 5 & 9 years	Cued and Free Recall Measures of Television Program		1. WMC-AVS vs. WMC-AS (cued; 5 years) 0.67 [0.35, 0.98] 2. WMC-AVS vs. WMC-AS (cued; 9 years) 0.23 [−0.08, 0.54] 3. WMC-AVS vs. WMC-AS (free; 5 years) 0.25 [−0.06, 0.56] 4. WMC-AVS vs. WMC-AS (free; 9 years) 0.64 [0.33, 0.96] 5. WMC-AVS vs. WMC-VS (cued; 5 years) 0.46 [0.14, 0.77] 6. WMC-AVS vs. WMC-VS (cued; 9 years) 1.20 [0.86, 1.53] 7. WMC-AVS vs. WMC-AS (free; 5 years) 0.12 [−0.19, 0.43 8. WMC-AVS vs. WMC-AS (free; 9 years) 0.79 [0.47, 1.12]
17	(Gillam et al., 1998)	To test the immediate recall of digits presented visually, auditorily, or audiovisually	Group differences	16	9.8 (1.2) 8–12 years	Audio-Visual Digit Recall Task		1. WMC-AVS vs. WMC-AS (WM) −0.26 [−0.95, 0.44] 2. WMC-AVS vs. WMC-AS (STM) −0.21 [−0.90, 0.49] 3. WMC-AVS vs. WMC-VS (WM) −0.19 [−0.89, 0.50] 4. WMC-AVS vs. WMC-VS (STM) 0.18 [−0.52, 0.87]
18	(Hatchette & Evans, 1983)	To determine whether audiovisual processing (temporal and spatial integration) is the critical factor in accounting for the performance on pattern matching tasks	Group differences	18	8.6 7.3–10 years	Multisensory temporal/spatial Pattern Matching Taks		1. WMC-AVS vs. WMC-AS (WM) 0.46 [−0.20, 1.12] 2. WMC-AVS vs. WMC-VS (spatial) 0.49 [−0.18, 1.16] 3. WMC-AVS vs. WMC-VS (temporal) −0.14 [−0.79, 0.51]
19	(Pillai & Yathiraj, 2017b)	To assess whether a correlation exists between two scoring of memory task (score and span) across three sensory conditions (auditory, visual, and auditory–visual)	Group differences	28	8.1 7–9 years	Visual and Audiovisual Scores and Span Tasks		1. WMC-AVS vs. WMC-AS (Score) 0.34 [−0.18, 0.87] 2. WMC-AVS vs. WMC-AS (Span) −0.44 [−0.97, 0.09] 3. WMC-AVS vs. WMC-VS (Score) 0.88 [0.33, 1.42 4. WMC-AVS vs. WMC-VS (Span) 0.46 [−0.07, 0.99]

Note. WM = working memory; VWM = visual working memory; AWM = auditory working memory; AV-MRTs = audiovisual motor reaction times; AMT = auditory memory task; ITPA = Illinois Test of Psycholinguistic Abilities; MS = multisensory; WISC–IV = Wechsler Memory Scale; McGurk = McGurk effect; ALT = Associative Learning Task; A-V = auditory–visual; V-A = visual–auditory; Total-WM = Total Score of VWM and AWM; DS = digit span; CMST = Complex Memory Span Task; RAMST = Revised Auditory Memory and Sequencing Test; CVLT-C = The California Verbal Learning Test for Children; WMC-AVS = Working Memory Capacity for Audiovisual Stimuli; WMC-AS = Working Memory Capacity for Auditory Stimuli; WMC-VS = Working Memory Capacity for Visual Stimuli; mo = Months; AVH/RM = Audiovisual Habituation Recognition Memory; VH/RM = Visual Habituation Recognition Memory; WM = working memory; STM = short-term memory.

**Table 5:** Characteristics and results of studies included in narrative synthesis.

Study Details				Participants		Measures		Results	
#	Citation	Aim of Study	Study Design	*N*	Age M (SD); Range	Multisensory Task/s	Memory Task/s	Correlation, *p*	Fishers’ z [95% CI]
1	(Broadbent et al., 2018)	To examine the role of multisensory information on incidental category learning during an attentional vigilance task	Correlation	185	8.17 (0.41) 6–10 years	Multisensory Attention Learning Task (MALT)	“AWM” WISC (Digit Span Backward)	*r* = 0.118, ns *r* = 0.009 ^a^, ns	0.12 [−0.03, 0.26] 0.01 [−0.14, 0.16]
2	(Cleary et al., 2001)	To examine the relationship between verbal digit span and each of the three different presentation conditions (visual, auditory and audiovisual)	Correlation	44	8.10 8–9 years	Auditory-plus-visual–spatial memory game	“AWM” WISC (Digit Span Forward &Backward)	*r* = 0.58, <0.01 *r* = 0.36, <0.05	0.66 [0.36, 0.97] 0.38 [0.07, 0.68]
3	(Barutchu et al., 2020)	See Table 2, study # 5 for further details				Associative Learning Tasks (ALT)	“AWM” WISC–IV WMI	*r* = 0.09, ns *r* = 0.17, ns	0.09 [−0.24, 0.42] 0.18 [−0.16, 0.50]

Note. AWM = auditory working memory; WISC–IV = Wechsler Memory Scale; WMI = Working Memory Index. ^a^ Digit Span Backward T score; ns = not significant.

#### 3.3.1. Participant Characteristics

A total of 1112 participants were drawn from the 21 studies, with 883 included for meta-analysis. Participant ages varied widely, ranging from 6 months to 15 years. However, the majority of studies (*n* = 18) focused on children aged 5 to 12 years, though the Denervaud et al. (2020b) study included children from 4 to 15 years, and two studies included infants (Choudhury et al., 2007; Ortiz-Mantilla et al., 2008).

#### 3.3.2. Task Characteristics in Correlational Studies

As there was considerable variability in the assessment protocol and how participants responded to stimuli related to multisensory tasks in the correlation studies, this meta-analysis conducted subgroup analyses focusing on either multisensory task responded to by MRTs or verbal multisensory tasks. In the multisensory MRTs subgroup, the majority (4/6) of studies utilized a Simple Audiovisual Detection Task (e.g., Alhamdan et al., 2023b; Barutchu et al., 2011; Barutchu et al., 2020; Denervaud et al., 2020b). Two further studies in children 6–12 years (Magimairaj et al., 2009; Montgomery et al., 2009) employed MRTs in color word recognition tasks, where participants were asked to make a simple decision (press the button) about which object matched the heard colour name. These multisensory MRT tasks were usually correlated with auditory and visual WM tasks including Digit Span Tasks (Forwards and Backwards), and Complex Memory Span Tasks (see Table 3 and Table 4 for details).

In general, the verbal response to multisensory tasks focused on assessing how individuals perceive and process simultaneously presented visual and auditory information and required verbal responses (e.g., Badian, 1977; Barutchu et al., 2019; Constantinidou et al., 2011; Crawford & Fry, 1979). For example, participants (aged 7–13) were asked to verbally identify associations between visual symbols displayed on a screen and corresponding auditory cues. Only one study (Davies et al., 2019) used a Change Detection paradigm requiring participants (aged 5–6) to determine whether the shape-sound pairing was the same or different. These verbal multisensory tasks were correlated with a wider range of auditory and visual WM tasks including Digit Span Tasks, Listening Recall Tasks, Odd-One Out Tasks, and the Auditory and Visual Sequential Memory subtests of the Illinois Test of Psycholinguistic Abilities (ITPA) (see Table 3 and Table 4 for details).

#### 3.3.3. Task Characteristics in Group Differences Studies

In group difference analysis, a single WM task was used with multi- and unisensory conditions, and effect sizes were extracted to assess the differences in WM capacity across three distinct modalities: audiovisual, auditory-alone, and visual-alone. In these tasks, participants (aged 5–12 years) were asked to verbally recall items (i.e., words or digits) presented under three conditions: (1) audiovisual, where the stimuli were presented simultaneously on a screen and verbalized, (2) auditory, where participants listened to verbalized names of objects or digits, and (3) visual, where participants were presented with visual objects or digits on a computer screen. Such tasks are generally considered as more complex than those used in correlation studies due to the involvement of multiple mental operations, including the need to integrate information from both auditory and visual modalities as well as the demands of encoding and verbal recall (see Table 5 for more details). Six of the 8 studies included in the group differences analyses involved school-age participants, while the two infants studies (Choudhury et al., 2007; Ortiz-Mantilla et al., 2008) assessed WM capacity in terms of looking time, i.e., in terms of visually driven attention (Goldberg & Wurtz, 1972) for two distinct modalities, i.e., audiovisual, and visual-alone. Further descriptions of the tasks are provided in Table 3.

### 3.4. Result of Bayesian Meta-Analyses

As previously mentioned, correlation data were grouped based on the type of assessment protocol of the multisensory task (multisensory MRTs, or verbal multisensory tasks). Following this, group difference data (i.e., effects sizes) were analysed to investigate the contribution of multisensory stimuli to WM capacity as compared to unisensory (auditory stimuli-alone and visual stimuli-alone). These categorisations were driven by the research aims and hypotheses of the review as well as to minimize result heterogeneity.

#### 3.4.1. Multisensory MRTs Tasks

**Figure 3:**
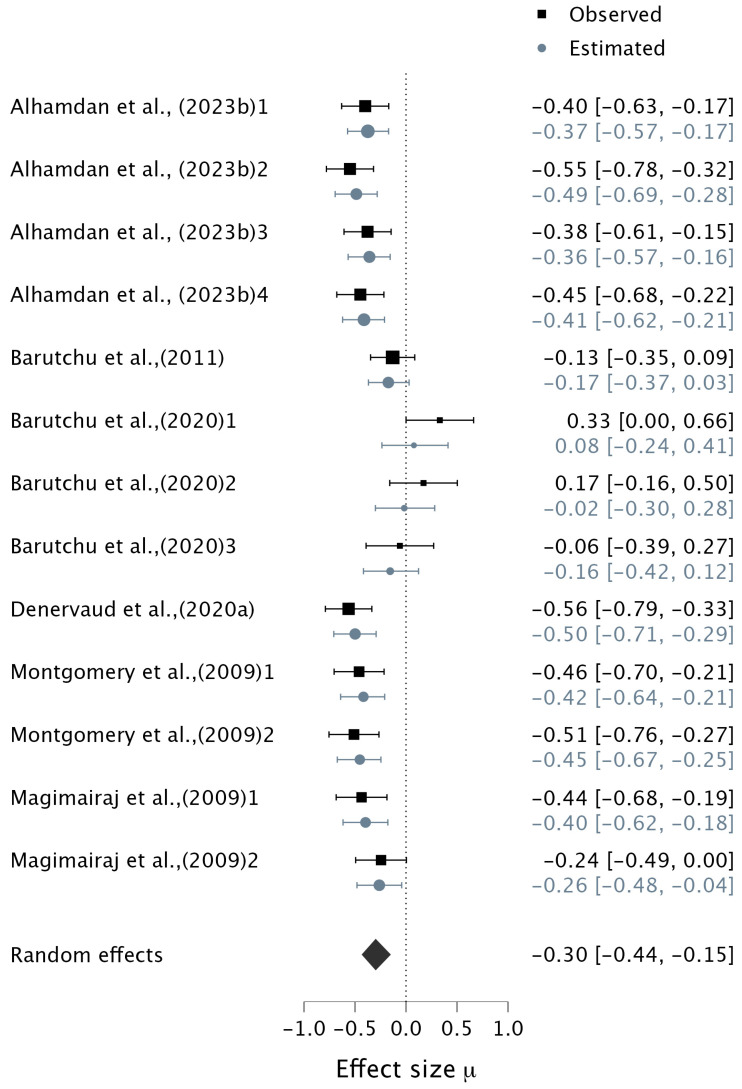
Forest plot showing pooled correlations (Fisher’s Z) for multisensory MRTs and auditory tasks as measures of WM capacity. Note. Observed per-study effect sizes (i.e., Fisher’s z) with 95% confidence intervals are shown in black. Estimated: the mean prior beliefs about the distribution per-study effect sizes and 95% credible intervals are shown in grey. All measures of WM capacity were auditory digits span, except that of (Alhamdan et al., 2023b), which included both visual and auditory digit span. (Source studies: Alhamdan et al., 2023b; Barutchu et al., 2011, 2020; Denervaud et al., 2020b; Magimairaj et al., 2009; Montgomery et al., 2009). Superscript numbers (1, 2, 3 & 4) after references in the fIgure indicate multiple effect sizes reported from the same source study (e.g., from different tasks, samples, or conditions).

Six studies contributed 13 datapoints to an analysis of MRTs. Four studies used the Simple Audiovisual Detection Task, where participants were required to press a button when they heard a sound (e.g., a beep), or saw a picture (e.g., flashing or a circle), or both. Two studies (Montgomery et al., 2009; Magimairaj et al., 2009) required participants to match a spoken colour name with the corresponding visual shape by pressing the appropriate button on a touchscreen as quickly as possible. Four studies contributed more than one datapoint (e.g., Alhamdan et al., 2023b, 4 datapoints; Barutchu et al., 2020, 3 datapoints; Magimairaj et al., 2009; Montgomery et al., 2009, 2 datapoints) (see Table 3 for details). In all six studies examining school-aged children with a mean age of 8 to 9 years, MRT tasks were employed, and auditory WM was tested alone except in one study (Alhamdan et al., 2023b) where both aspects of auditory and visual Digit Span were used to assess WM capacity. The results of the Bayesian meta-analysis indicated strong evidence for the relationship between MRT and both auditory and visual WM, in favour of the alternative hypothesis (pooled correlation: μ = −0.30, 95% CI [−0.44, −0.14], BF_10_ = 26.84) (Figure 3). However, there was extreme evidence of heterogeneity (τ = 0.22, BF_10_ = 227.06, 95% CI [0.10, 0.38]).

#### 3.4.2. Verbal Multisensory Tasks

**Figure 4:**
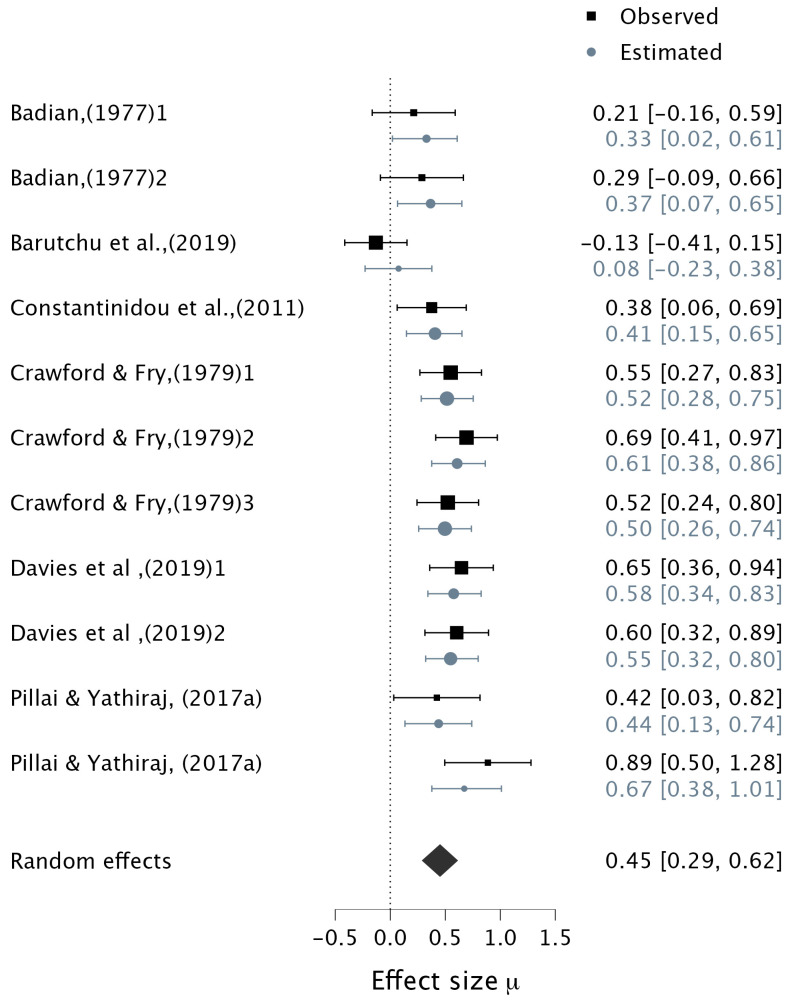
Forest plot showing pooled correlations (Fisher’s Z) for verbal multisensory measures and a variety of auditory and visual tasks as measures of WM capacity. Note. Observed per-study effect sizes (i.e., Fisher’s z) with 95% confidence intervals are shown in black. Estimated: the mean prior beliefs about the distribution per-study effect sizes and 95% credible intervals are shown in grey. (Source studies: Barutchu et al., 2019; Badian, 1977; Crawford & Fry, 1979; Constantinidou et al., 2011; Davies et al., 2019; Pillai & Yathiraj, 2017a). Superscript numbers (1, 2 & 3) after references in the figure indicate multiple effect sizes reported from the same source study (e.g., from different tasks, samples, or conditions).

Six studies contributed 11 datapoints to an analysis of verbally assessed multisensory tasks (see Table 3). Tasks were variable in all of these studies: Five studies required participants to verbalize the audiovisual stimuli (see Table 5 for details) (e.g., Badian, 1977; Barutchu et al., 2019; Constantinidou et al., 2011; Crawford & Fry, 1979; Pillai & Yathiraj, 2017a), and one study (Davies et al., 2019) employed a change detection audiovisual task in which participants were instructed to verbally respond ‘same’ or ‘different’ depending on whether the audiovisual pairing on the test display was the same as an old pairing. All six studies examined school-aged children from 5- to 13-years. Bayesian meta-analysis indicated extreme evidence supporting the alternative hypothesis for the association between verbal multisensory tasks and both visual and auditory WM (pooled correlation: μ = 0.45, 95% CI [0.29, 0.62], BF_10_ = 337.91) (Figure 4). There was very strong evidence of heterogeneity (τ = 0.21, BF_10_ = 33.77, 95% CI [0.08, 0.41]).

Thus, the meta-effect demonstrates that, while there is some degree of variability across studies, there is extreme evidence showing that verbal semantic multisensory tasks requiring adequate receptive language and verbal memory had a stronger association with the verbally assessed WM tasks than with MRT tasks. This likely to reflect the different cognitive demands of MRT versus verbal multisensory tasks, and may also support the existence of distinct developmental mechanisms and or more likely developmental timetables as suggested by recent brain imaging studies (Gunaydin et al., 2025). The variability in MRT outcomes may also be influenced by age-related changes in motor speed and executive control, whereas verbal tasks which involve integrated sensory, linguistic, and memory processes, may be more sensitive indicators of WM development in educational contexts.

#### 3.4.3. The Contribution of Multisensory vs. Unisensory Stimuli to WM Capacity

As some studies compared WM capacity for multisensory stimuli within a single task, we analyzed the overall effect size for the contribution of multisensory stimuli to WM capacity in comparison to auditory- alone and visual- alone stimuli. Eight studies contributed 29 datapoints for inclusion in this meta-analysis. Six studies (Constantinidou et al., 2011; Constantinidou & Evripidou, 2012; Field & Anderson, 1985; Gillam et al., 1998; Hatchette & Evans, 1983; Pillai & Yathiraj, 2017b) required school-age participants (5–13 years) to verbally recall the presented stimuli (either items or digits), whilst two preverbal infant studies of children aged 6–9 months (Choudhury et al., 2007; Ortiz-Mantilla et al., 2008) employed a looking-time task involving only two distinct modalities: audiovisual and visual-alone to measure auditory–visual habituation/recognition memory tasks (see Table 3 for more details).

**Figure 5:**
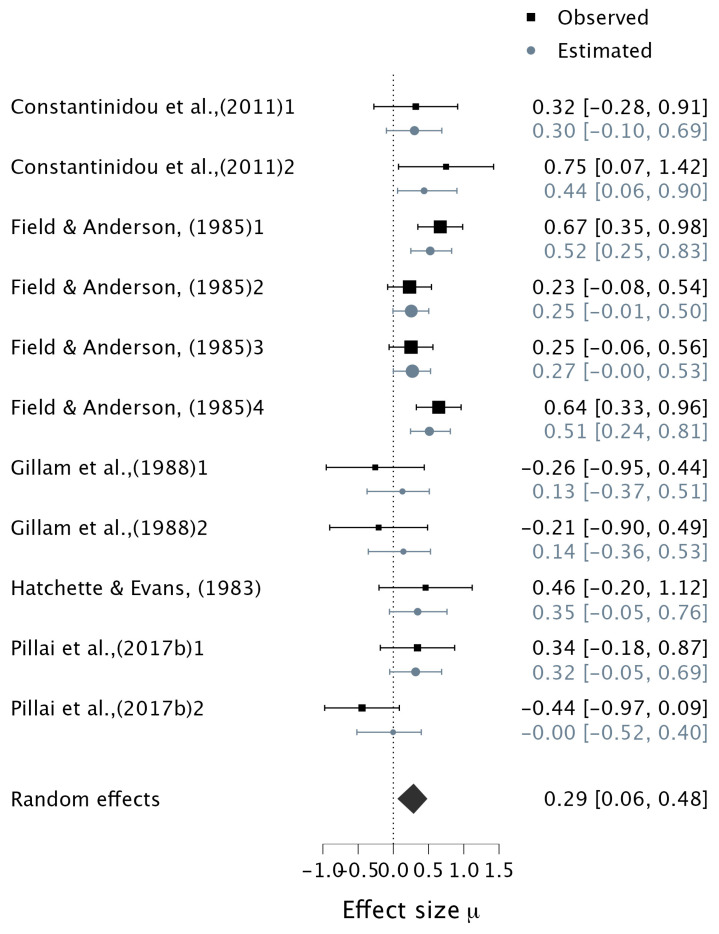
Forest plot showing pooled effect sizes (Cohen’s *d*) for the response to multisensory stimuli vs. auditory stimuli to WM capacity. Note. Observed per-study effect sizes (i.e., Cohen’s *d*) with 95% confidence intervals are shown in black. Estimated: the mean prior beliefs about the distribution per-study effect sizes and 95% credible intervals are shown in grey. (Source studies: Constantinidou & Evripidou, 2012; Constantinidou et al., 2011; Field & Anderson, 1985; Gillam et al., 1998; Hatchette & Evans, 1983; Pillai & Yathiraj, 2017b). Superscript numbers (1, 2, 3 & 4) after references in the fIgure indicate multiple effect sizes reported from the same source study (e.g., from different tasks, samples, or conditions).

For the contribution of audiovisual stimuli versus auditory-only stimuli to WM capacity, Bayesian analysis showed moderate evidence of the alternative hypothesis, with a small effect (pooled correlation: μ = 0.29, 95% CI [0.08, 0.48], BF_10_ = 3.56) (Figure 5). There was moderate evidence of heterogeneity (τ = 0.22, BF_10_ = 3.72, 95% CI [0.05, 0.47]).

**Figure 6:**
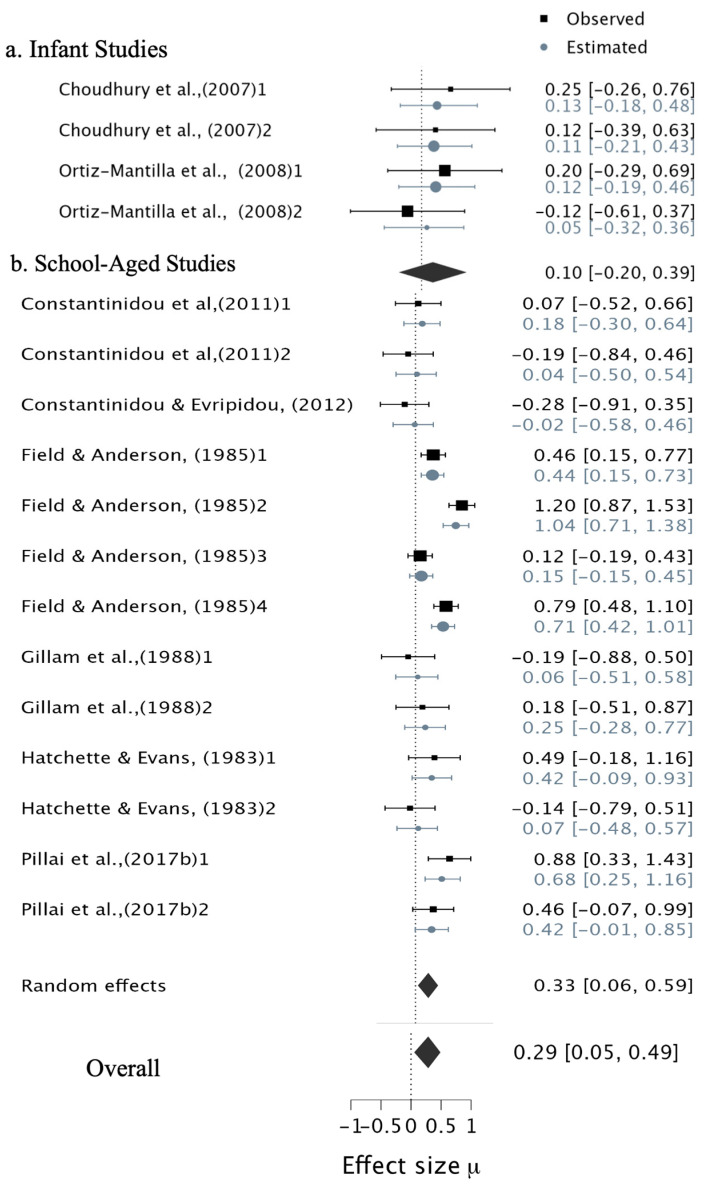
Forest plot showing pooled effect sizes (Cohen’s *d*) for the response to multisensory stimuli vs. visual stimuli to WM capacity. Infant studies assessed WM in terms of looking time that has been known to correlate and reflect shift with visually driven attention (Goldberg & Wurtz, 1972). (Source studies: Choudhury et al., 2007; Constantinidou & Evripidou, 2012; Constantinidou et al., 2011; Field & Anderson, 1985; Gillam et al., 1998; Hatchette & Evans, 1983; Ortiz-Mantilla et al., 2008; Pillai & Yathiraj, 2017b). Superscript numbers (1, 2, 3 & 4) after references in the fIgure indicate multiple effect sizes reported from the same source study (e.g., from different tasks, samples, or conditions).

For the contribution of audiovisual stimuli versus visual-only stimuli to WM capacity, the Bayesian results of the overall effect size showed moderate evidence supporting the alternative hypothesis, with a small effect (pooled correlation: μ = 0.29, 95% CI [0.07, 0.49], BF_10_ = 3.06) (Figure 6; overall effect sizes). However, there was extreme evidence of heterogeneity (τ = 0.35, BF_10_ = 23,100, 95% CI [0.20, 0.56]).

Given the variability in the range of ages across studies (including two infant studies and the remainder involving school-age children), and the use of different measurement techniques, subgroup analyses were conducted. For infants studies (Choudhury et al., 2007; Ortiz-Mantilla et al., 2008) ‘looking time’ was used as the outcome, and results showed no evidence of differences between WM capacity for AVS compared to vs. in infants group (pooled correlation: μ = 0.10, 95% CI [−0.19, 0.40], BF_10_ = 0.21), with low heterogeneity (τ = 0.14, BF_10_ = 0.51) (Figure 6a). For school-aged children (5–13 years), when tasks required participants to verbally recall the presented stimuli (either items or digits), we found anecdotal evidence supporting the alternative hypothesis for differences between WM capacity for audiovisual stimuli compared to visual-only stimuli, with a medium effect (pooled correlation: μ = 0.33, 95% CI [0.06, 0.58], BF_10_ = 2.47). However, there was extreme heterogeneity (τ = 0.39, BF10 = 12,075.37) (Figure 6b).

### 3.5. Moderator Meta-Regression Analysis

Meta-regression analysis using traditional meta-analysis was conducted to explore whether accounting for mean age of participants as a moderator could explain the heterogeneity observed within subgroups. Results showed that the predictor variable “mean age” had a statistically significant effect on the correlation analysis between multisensory MRTs, verbal multisensory tasks, and visual and auditory WM (z = 5.66 *p* < 0.001, z = −2.59 *p* = 0.01, respectively). Heterogeneity was very low (τ^2^ = 8.329 × 10^−6^, *Q* (11) = 11.04, *p* = 0.44, *I*^2^ = 0%) in the first subgroup (i.e., multisensory MRTs), while there was moderate heterogeneity in the second subgroup (verbal multisensory tasks) (τ^2^ = 0.02, *Q* (9) = 16.62, *p* = 0.05, I^2^ = 45%). However, there was no statistically significant effect of the mean age on the group differences analysis (i.e., the contribution of multisensory vs. unisensory stimuli to WM capacity), with moderate heterogeneity (I^2^ 65% −69%). I^2^ values were interpreted based on the guidelines by Higgins and Thompson (2002). Finally, we also included τ^2^ and the Q statistics as additional checks of heterogeneity (Borenstein et al., 2021).

### 3.6. Results of Narrative Synthesis

Two additional studies (Broadbent et al., 2018; Cleary et al., 2001) and two datapoints from Barutchu et al. (2020) met the inclusion criteria for this systematic review and provided correlation data, but the tasks used in these studies did not fit within the predefined categories of MRTs or verbal non motor multisensory tasks (i.e., Motor non-timed tasks (i.e., tasks involving manual motor actions but focusing on the accuracy of responses rather than response time, as defined in Section 2.5), thus these studies were not included in the meta-analysis. They are instead briefly summarized below (see Table 4).

Two of the three studies (Barutchu et al., 2020; Broadbent et al., 2018) employed Multisensory Learning Tasks, and required participants to respond to specific target stimuli by pressing the appropriate button. In particular, Barutchu et al. (2020) used an Associate Learning Task, where participants were required to learn associations between visual symbols and auditory sounds, while Broadbent et al. (2018) used a Multisensory Attention Learning Task (MALT), where the focus was on how sensory cues (visual, auditory, or combined) affect attention and learning, particularly in the context of animal categorization. Results across the two studies showed no correlation between multisensory learning tasks and digit span tasks. Lastly (Cleary et al., 2001) used a Multisensory Spatial Memory Game, where participants were asked to press appropriate buttons on a response box when presented with sounds (colour names) and or flashing buttons. Results of this study showed better more accurate performance on the multisensory spatial memory game that significantly correlated with both forward and backward digits. Importantly, all tasks only measured accuracy of response rather than speed of responses (i.e., how fast participants can identify the stimuli). Results of these studies were variable and inconclusive (see Table 4 for all correlation values). Further description of multisensory tasks can be found in Table 5.

To summarize, our results showed that verbal multisensory tasks were more strongly and consistently associated with verbally assessed auditory and visual WM than motor reaction time (MRT) tasks, suggesting distinct cognitive demands and developmental trajectories. The meta-analyses also revealed a modest advantage of multisensory over unisensory stimuli in supporting WM capacity. Moderator analysis indicated that age significantly influenced outcomes within both MRT and verbal multisensory subgroups, but not in group difference effects. Finally, studies that did not align with the predefined task categories were narratively reviewed and showed mixed, inconclusive results.

## 4. Discussion

The current systematic review and Bayesian meta-analyses aimed to assess the current literature examining the association between multisensory processing, including motor and verbal multisensory tasks, and measures of auditory and visual WM in children up to 15 years nothing that there is very little consideration of attention and WM in preverbal children (Teller et al., 1986; Teller, 1997). In fact, there are only a few mesures of timing response to either visual auditory or multisensory in infants expects for the two included studies (Choudhury et al., 2007; Ortiz-Mantilla et al., 2008) that investigated infant attention in terms of looking time. Based on the assumption that visual attention correlates with WM as in primates and older children, the review also aimed to explore the contribution of multisensory stimuli to WM capacity compared to unisensory auditory-only or visual-only stimuli in the same post verbal population. A total of 21 studies of moderate to high quality met the inclusion criteria, and 19 studies contributed to the correlation or group difference meta-analyses.

Findings from this review provide three main insights.

1. First, verbal multisensory processing was extremely/decisively associated with visual and auditory WM in children, while non-verbal motor reaction time multisensory tasks showed strong correlations with auditory and visual WM, suggesting verbal and MRTs multisensory processing contribute differently to WM performance, and might follow different developmental trajectories as suggested by recent brain imaging study (Gunaydin et al., 2025; Sydnor et al., 2025).
2. Furthermore, as expected, age was a significant moderator for the association between multisensory processing and visual and auditory WM, highlighting both the developmental maturation of cognitively complex WM and multisensory processing abilities.
3. Lastly, we found strong evidence that WM capacity for both verbal and MRT multisensory stimuli was higher than either visual or auditory stimuli alone. These results will first be discussed with reference to age and development, followed by the association between MRTs and verbal multisensory tasks and WM, and finally the contributions of multisensory stimuli to WM capacity will be considered.

### 4.1. Age Development in Motor and Verbal Multisensory Tasks and Their Relationship with WM

The previously noted a behavioural developmental study conducted by Tremblay et al. (2007) suggested that non-speech (MRT) and verbal aspects of multisensory processing may be dissociable and exhibit different developmental stages. This suggestion is supported by neuroimaging studies showing that in both adults and children, the site of activation of neurons during audiovisual processing varies based on the nature of the stimuli—verbal or nonverbal (Gkintoni et al., 2025; Hocking & Price, 2009; Thierry & Price, 2006).

Our findings of extreme/decisive evidence for an association between verbal multisensory tasks and visual and auditory WM, as opposed to a weak, though significant, associations between MRTs and visual and auditory WM, partially support a task function distinction and fit well with previous psychophysical and imaging studies (Hocking & Price, 2009; Okada & Hickok, 2009; Tremblay et al., 2007). This motor time dissociation has also been demonstrated well elsewhere, as both verbal and motor multisensory tasks have been shown to support cognitive functions such as learning and WM (Alhamdan et al., 2023b; Fifer et al., 2013; Heikkilä et al., 2015; Heikkilä & Tiippana, 2016). Such sensori-motor development with age is well known to follow different temporal trajectories and neural pathways both in terms of time and accuracy (Brandwein et al., 2011; Nardini et al., 2016). As noted earlier, simple hand motor function appears in infants at 4–5-months, yet there is much later maturation of the complex inter-relating motor pathways and muscles of diaphragm, throat, esophagus, tongue, lips, jaws, and cheeks, associated with sound production and the emergence of deliberate/conscious oral verbalization starting around 12-months (Chartier et al., 2018; Dionne-Dostie et al., 2015). Prior research has also shown that the time needed to access the verbal lexicon store necessary to allow individuals to retrieve and manipulate linguistic information is greater than time for motor responses alone (Bates et al., 1986; Bucci & Freedman, 1978). Furthermore, our findings highlight that the capacity to integrate multisensory information from different sensory sources, especially for verbal material, may enhance the efficient usage of information in more complex WM tasks. Such findings are consistent with the idea that multisensory integration of letter-speech sounds is associated with reading ability (Blau et al., 2010; Snowling et al., 2022), and the development of fluent reading skills (Bradley & Bryant, 1983; Elhassan et al., 2017; Froyen et al., 2011) in primary school grades. Further, Massaro et al. (1986) found a positive correlation in children between lip-reading ability and multisensory speech information, suggesting that lip-reading performance for audiovisual speech using McGurk effect reaches adult like levels sometime after the child’s sixth year (Massaro et al., 1986). Despite these insights, a significant developmental gap remains in the behavioural literature, particularly concerning children aged 2–4 years—a critical period for the emergence of receptive and expressive verbal language, multisensory processing and early WM capacity and time when testing compliance is difficult to maintain and an experimenter cannot be certain a child understands the task instructions of request or wishes to co-operate. Hence, Future studies should adopt age-appropriate, play-based paradigms using nonverbal cues (e.g., eye-tracking or preferential looking) to investigate these preverbal processes often on hand held device. Additionally, translating such findings into practice requires more targeted recommendations requiring audiovisual technology and devices for both teaching of motor skills and verbal language measurement. For example, using audiovisual cues to support early language comprehension in toddlers or differentiated multisensory instruction based on WM profiles in primary-aged children. Such efforts would enhance both the theoretical and applied contributions of multisensory research in developmental contexts.

Considering the growing evidence linking multisensory processing to cognitive development, practical integration into home and educational settings is both necessary and timely. Effective multisensory teaching strategies in infants include pairing spoken words with visual and tactile stimuli, then in early school age incorporating tactile ma-terials into reading and math instruction, and using interactive technologies that engage multiple senses simultaneously (Cuturi et al., 2022; Mathias et al., 2022; Shams & Seitz, 2008). These methods align with findings that multisensory input enhances neural effi-ciency i.e., the brain’s ability to process information more effectively (Park & Reu-ter-Lorenz, 2009) and learning outcomes, particularly in tasks that require or assess working memory and attention (Barutchu et al., 2020; Davies et al., 2019). Moreover, differentiated instruction based on developmental stages—for example, using more concrete sensory cues in early childhood and abstract multimodal tasks in later years can help accommodate individual learning profiles (Alhamdan et al., 2023b; Bremner et al., 2012). Embedding such strategies into everyday classroom activities holds promise for improving academic performance and supporting neurodiverse learners (Dobinson & Dockrell, 2021; Yi & Heidari Matin, 2025).

As alluded to above, the results from the various types of multisensory tasks, especially in the domains of verbal responses and motor processing, indicate varying strengths of correlations between performance of multisensory tasks and accuracy of visual and auditory WM performance, while also highlighting that different types of tasks and the requisite battery of cognitive skills to accomplish the task can affect research findings e.g., ability to articulate particularly long words (Gathercole & Baddeley, 1990). Indeed, all the tasks included in our meta-analysis required a range of other cognitive abilities, including the ability to attend to, process, and respond to the instructions, with an adequate level of receptive and expressive language skills needed to understand instructions prior to responding either verbally or with motor nonverbal responses. Thus, future research needs to consider these different aspects of multisensory tasks as well as a child’s abilities in receptive and expressive language and motor capability when assessing the association between multisensory processing and cognitive development.

In addition, our hypothesis that age and the rapid and extensive brain growth and significant changes during infancy and early childhood (Christian et al., 2017; Hedman et al., 2012) associated brain function should significantly moderate the association between multisensory tasks for both verbal and MRTs was well supported by our meta-regression. This is consistent with much previous research showing age-related improvement in multisensory processing (Barutchu et al., 2019; Brandwein et al., 2011) and cognitive abilities such as attention and visual WM (Buss et al., 2014; Buss et al., 2018). Indeed, our own research has demonstrated age-related improvements in multisensory MRTs that become faster and more efficient in older children, and then become slower in older adults later in life (Ebaid & Crewther, 2020); multisensory MRTs are also associated with better WM performance in both visual and auditory tasks (Alhamdan et al., 2022, 2023a, 2023b). Equally important is the fact that knowledge and experience may also provide further explanation for age-related improvement. Specifically, with the maturation of children, the growth of knowledge in using processing strategies and increasing familiarity with the task are considered essential factors contributing to the development of both multisensory and the visual and auditory aspects of WM (Anderson & Wagovich, 2010; S. J. Pickering, 2001; Stokes & Bors, 2001).

### 4.2. Contribution of Multisensory Stimuli to WM Capacity

In addition to examining the associations between multisensory tasks (MRTs and verbal) and visual and auditory WM, this review considered how WM capacity may improve when presented with multisensory stimuli compared to unisensory visual or auditory stimuli alone. Notably, tasks used in the group difference analyses in 6 out of 8 studies were considered more complex than those used in correlation meta-analyses due to the involvement of multiple mental operations, which required participants to integrate information from both auditory and visual modalities, retain this information in memory (i.e., items or digits), and orally recall the items to successfully complete articulation of such verbally based tasks. Overall, our findings demonstrated moderate evidence that multisensory stimuli contributed significantly to WM capacity more than unisensory visual or auditory stimuli alone. This suggests that more information can be retained and utilized with WM when children are provided with input from multiple senses. This finding is consistent with several past studies that have demonstrated WM capacity is usually greater for audiovisual stimuli than unimodal stimuli in both adults and children aged 7 to 13 years (Heikkilä et al., 2015; Heikkilä & Tiippana, 2016).

Although the contribution of age was not a significant moderator of these group difference analyses, the subgroups analyses (infants and school-age children) suggest that age-related effects do occur, given that in infant studies (Choudhury et al., 2007; Ortiz-Mantilla et al., 2008) the results indicated that both multisensory and visual-only stimuli showed comparable/similar WM attentional performances. However, our findings from school-age children did show *anecdotal* evidence of differences in the contributions of multisensory stimuli to both auditory and visual WM. Whilst previous studies have suggested that multisensory facilitation (i.e., matching and transferring information across senses) appears early in development of young infants (D. J. Lewkowicz & Lickliter, 1994; Bahrick & Lickliter, 2012; Flom & Bahrick, 2007; G. Lewkowicz, 2009), we did not observe any differences between multisensory and visual stimuli-alone, although this finding may be due to the limited number of studies included in the infant subgroup, which necessarily only included pre-verbal infants. Furthermore, there is evidence that the actual combination and integration of multisensory stimuli appears to develop later in childhood (Alhamdan et al., 2022; Barutchu et al., 2009; Brandwein et al., 2011). This is likely because these processes are dependent on the maturation of cortical brain regions and attention capabilities which continue to develop throughout later childhood and early adolescence (Corbetta & Shulman, 2002; Kanaka et al., 2008; Mabbott et al., 2006; Smith & Chatterjee, 2008). Importantly, however, the inclusion of preverbal infant studies alongside those with older children shows a conceptual gap in maturity that must be acknowledged but not the total lack of such neural and cognitive mechanisms in infant audiovisual processing (e.g., perceptual binding, attentional orienting of young infants’ eyes (first few days), mouth towards nipple for drinking and head (6–8 weeks and to mother’s voice few days). A considerable body of literature relating to visually driven eye movements and attention and multisensory (visual to auditory sounds) very early in life fundamentally the same but less accurate from the executive and memory-based processes underlying WM performance in school-aged children. Thus, although both age groups engage in multisensory processing, the functions and outcomes of that processing differ in efficiency substantially, they should be interpreted as points along a single developmental continuum. Future research should aim to better define the temporal trajectory of these mechanisms preverbally, possibly by adopting age old ‘looking time” and habituation (Horowitz et al., 1972) and animal research (Goldberg & Wurtz, 1972) with preverbal monkeys’ complementary eye movement measures of attention shifting and looking behaviours early neurophysiological and behavioural approaches tailored to the developmental stage. Taken together, these results show that age-related differences may explain the different results between infants and school-age children in the context of brain development and all forms of cognition including multisensory WM. Furthermore, considering the limited number of studies considering the development of multisensory processing during WM tasks in children, further research using psychophysics and both electrical and magnetic brain imaging is needed in this area to gain a more comprehensive understanding of both anatomical and functional age-related factors. Moreover, as the visually presented items in many of these tasks (e.g., digits, coloured stimuli) can be readily verbalized, it remains unclear whether the children, who are acquainted with the objects, employed additional verbal encoding (i.e., multisensory processing) to enhance their performance on visual-only WM tasks. Therefore, future research should address the potential confound of potentially verbalizable visual stimuli on the assessment of multisensory WM.

### 4.3. Limitations and Future Direction

The strength of this review has been the use of Bayesian probability statistics in accordance with recent analytical recommendations (Bartoš et al., 2021; Bartoš et al., 2023) to provide robust assessments of the strength of evidence for the alternative hypothesis. This approach was employed in two different analyses (correlational and group differences Bayesian meta-analysis) to enhance the precision and depth of our investigation into the association and contributions of multisensory stimuli to WM performance. However, there are several limitations of the literature we reviewed that also need to be considered. First, there were very few studies available for inclusion involving infants (only 2 out of 21), and none included children aged 2- to 4-years who were also noticeably absent from our meta-analysis. Consequently, the developmental trajectory in this critical early period remains understudied, and further investigation is needed to investigate the effects of multisensory processing to cognitive development, such as WM and intelligence, during these formative years. Since our review included studies with a wide age range, we attempted to address potential age-related effects by using the mean age of participants as a moderator. However, it is crucial to acknowledge that mean age might not provide a complete representation of the sample within each study, particularly those that cover a large age range (e.g., Denervaud et al., 2020b [4–15-year-olds]). Therefore, more robust research designs, with prior consideration for intelligence (both verbal and non-verbal) and working memory ability at several time points, are needed to determine age-related changes and understanding of the longitudinal developmental trajectory of multisensory and WM relations.

Furthermore, the scarcity of studies that measured visual WM in our review presents another severe limitation given that vision is recognized as the primary information channel of the primate brain (H. E. Pickering et al., 2022). Most included studies exclusively focussed on traditional auditory-verbal WM digit tasks, in line with the prominent multicomponent view of language as the basis of WM as a cognitive psychological issue (Baddeley, 2007, 2012 and the WISC and WAIS tests), while giving comparatively little consideration to more primate like visual-based WM. However, electrophysiological and brain imaging studies have consistently shown that vision drives attention and eye movements, and that the parieto-frontal networks of the dorsal visual stream of the brain carry both spatial and temporal information fastest and hence dominate attention and working memory manipulations from the time of birth (e.g., Johnson, 1990; Nelson, 1995; Laycock et al., 2022). Therefore, including assessments of both visual and auditory WM would provide a more comprehensive view of how multisensory processing affects different aspects of WM. Additionally, the visually presented digit span tasks employed in some of the included studies could be easily verbalized, meaning children may have employed additional verbal encoding to enhance their task performance, which may confound the ability to accurately compare visual and multisensory WM abilities. Thus, it will be important for future research considering visual WM to also include tasks with stimuli that are less conducive to verbalization, as this will help to minimize unintentional multisensory processing for visual tasks. Moreover, other individual and contextual factors such as socioeconomic status, bilingualism, lip reading (Massaro et al., 1986) and cultural variation in sensory processing were rarely considered in the included studies. These variables may influence attentional control, language processing, and familiarity with task stimuli, and should be accounted for in future research to enhance the ecological validity and generalizability of findings across diverse populations.

### 4.4. Conclusion and Implications

This current review aimed to explore whether multisensory processing using different types of tasks (verbal and MRTs) contributes to visual and auditory WM performance. Overall, our findings provide preliminary insight into the impact of multisensory tasks such as verbal and motor RT tasks that contribute differentially to WM performance. The main findings from our Bayesian meta-analysis revealed that multisensory processing (assessed verbally) was decisively associated with visual and auditory WM, while MRTs of multisensory processing also showed a strong association with visual and auditory WM tasks. These findings suggest that performances on verbal and motor multisensory tasks contribute differentially to WM performances, which supports observations that these abilities appear to have different developmental trajectories, presumably due to how motor and visuomotor function develops in childhood (Alhamdan et al., 2022, 2023a, 2023b). Furthermore, our results confirm that multisensory stimuli, when compared to unisensory stimuli, resulted in a higher WM capacity in school-aged children and adolescents, suggesting that multisensory information may enhance retention or manipulation of information in mind. Additionally, results from our current review also indicate that age is an important factor that moderates the association between multisensory tasks and visual and auditory WM, contributing to the expected increase in cognitive abilities, and the faster and more efficient multisensory processing seen with age. However, it is important to note that the consideration of other factors, such as years of schooling, intellectual ability, and language skills, is crucial for future studies and requires further investigation. This review has also highlighted the dearth of evidence regarding the development of such processes and abilities in pre expressively verbal toddlers aged 2 to 4 years of age.

This review also has important implications for cognitive and neuropsychological assessment of motor and speech-based tasks, in general terms of brain function and in school-based use of multisensory information in learning methods and educational activities. For example, the use of multisensory information could prove highly advantageous in enhancing learning outcomes, particularly where information retention is crucial. Moreover, this review highlighted the potential importance of multisensory processing to WM and learning skills in neurotypical children; however, it is important to acknowledge that multisensory teaching strategies may also more particularly be employed to support students with neurodevelopmental disorders such as those with learning difficulties such as dyslexia (Eroğlu et al., 2022; Hahn et al., 2014; Nijakowska, 2008, 2013) and autism spectrum disorder (ASD) (Molholm et al., 2020) or mutism. Specific investigation in these populations is necessary in terms of future educational and psychological research.

## Figures and Tables

**Table 1:** Sample MEDLINE (Ovid) search strategy.

No	Search Term (S)
1.	(sound* ADJ2 (vision OR visual OR sight*)).mp
2.	(multisensory ADJ2 (integration OR perspective OR processing)).mp
3.	(sensory ADJ2 (integration OR cross-modal OR multiple)).mp
4.	Intersensory processing/OR sensory integration/
5.	((Audiovisual OR audio-visual) ADJ2 (synchrony OR information)).mp
6.	Visual auditory integration.mp
7.	Visual perception.mp OR exp Visual perception/
8.	Auditory perception.mp OR exp Auditory perception/
9.	7 and 8
10.	1 or 2 or 3 or 4 or 5 or 6 or 7 or 8 or 9
11.	((working OR short-term OR long-term OR verbal OR visual OR spatial) ADJ2 memory*).mp
12.	memory/ or long-term memory/ or short-term memory/ or spatial memory/ or verbal memory/ or visual memory/
13.	11 or 12
14.	(Child* ADJ2(development OR early OR school age* OR pre-school OR young)).mp
15.	((preschool OR pre-school OR school) ADJ2 student*).mp
16.	Birth OR infant OR toddler OR newborn OR child*
17.	elementary school students/or preschool students/childhood development/or early childhood development/exp Infant Development/
18.	14 or 15 or 16 or 17
19.	10 and 13 and 18

Note. ADJ is a proximity operator that finds words within a certain distance of each other. ADJ2 finds each word within 2 words of the other. This can be represented differently in different databases.

## Supplementary Materials

The following supporting information can be downloaded at: https://www.mdpi.com/article/10.3390/ejihpe15080157/s1, Figure S1: PsyINFO (Ovid) Database; Figure S2: MEDLINE (Ovid) Database; Figure S3: EMBASE (Ovid) Database; Figure S4: CINAHL (EBSCO) Database; Figure S5: PubMed Database; Figure S6: Web of sciences (ISI) Database; Table S1: PRISMA Checklist (Page et al., 2021); Table S2: Summary of Amendments to PROSPERO Protocol (CRD42020148110; Alhamdan et al., 2019) (D. J. Lewkowicz & Lickliter, 1994; Barutchu et al., 2009; Brandwein et al., 2011; Nardini et al., 2008; JASP Team, 2022); Table S3: Search Strategy for all Databases; Table S4: Complete data extractions, including means, N, and SD for each group to calculate effect sizes for group difference studies.
