# Supplementary material for: From Senses to Memory During Childhood: A Systematic Review and Bayesian Meta-Analysis Exploring Multisensory Processing and Working Memory Development
Source: Eur J Investig Health Psychol Educ. 2025 Aug 12;15(8):157. doi: 10.3390/ejihpe15080157 (PMC12385695; doi:10.3390/ejihpe15080157)
Supplement: Supplementary file 1 [file ejihpe-15-00157-s001.zip › ejihpe-3629724-supplementary.pdf]

Supplementary Material

From Senses to Memory During Childhood: A Systematic Review and Bayesian Meta-Analyses Exploring Multisensory Processing and Working Memory Development

Areej A. Alhamdan*, Hayley E. Pickering, Melanie J. Murphy& Sheila G. Crewther

Supplementary Table S1. PRISMA Checklist

**TITLE**			
Title	1	Identify the report as a systematic review.	1
**ABSTRACT**			
Abstract	2	See the PRISMA 2020 for Abstracts checklist.	2
**INTRODUCTION**			
Rationale	3	Describe the rationale for the review in the context of existing knowledge.	3 – 8
Objectives	4	Provide an explicit statement of the objective(s) or question(s) the review addresses.	9 –10
**METHODS**			
Eligibility criteria	5	Specify the inclusion and exclusion criteria for the review and how studies were grouped for the syntheses.	12 – 13
Information sources	6	Specify all databases, registers, websites, organisations, reference lists and other sources searched or consulted to identify studies. Specify the date when each source was last searched or consulted.	10 –11 &Table 1
Search strategy	7	Present the full search strategies for all databases, registers and websites, including any filters and limits used.	11 & Figure S1 to S6
Selection process	8	Specify the methods used to decide whether a study met the inclusion criteria of the review, including how many reviewers screened each record and each report retrieved, whether they worked independently, and if applicable, details of automation tools used in the process.	12 – 13
Data collection process	9	Specify the methods used to collect data from reports, including how many reviewers collected data from each report, whether they worked independently, any processes for obtaining or confirming data from study investigators, and if applicable, details of automation tools used in the process.	12 –1 5
Data items	10a	List and define all outcomes for which data were sought. Specify whether all results that were compatible with each outcome domain in each study were sought (e.g. for all measures, time points, analyses), and if not, the methods used to decide which results to collect.	14 –16
	10b	List and define all other variables for which data were sought (e.g. participant and intervention characteristics, funding sources). Describe any assumptions made about any missing or unclear information.	14 –17
Study risk of bias assessment	11	Specify the methods used to assess risk of bias in the included studies, including details of the tool(s) used, how many reviewers assessed each study and whether they worked independently, and if applicable, details of automation tools used in the process.	13 –14
Effect measures	12	Specify for each outcome the effect measure(s) (e.g. risk ratio, mean difference) used in the synthesis or presentation of results.	14 –-16
Synthesis methods	13a	Describe the processes used to decide which studies were eligible for each synthesis (e.g. tabulating the study intervention characteristics and comparing against the planned groups for each synthesis (item #5)).	14 –16
	13b	Describe any methods required to prepare the data for presentation or synthesis, such as handling of missing summary statistics, or data conversions.	16 –18
	13c	Describe any methods used to tabulate or visually display results of individual studies and syntheses.	17–18
	13d	Describe any methods used to synthesize results and provide a rationale for the choice(s). If meta-analysis was performed, describe the model(s), method(s) to identify the presence and extent of statistical heterogeneity, and software package(s) used.	18
	13e	Describe any methods used to explore possible causes of heterogeneity among study results (e.g. subgroup analysis, meta-regression).	17–18
	13f	Describe any sensitivity analyses conducted to assess robustness of the synthesized results.	18
Reporting bias assessment	14	Describe any methods used to assess risk of bias due to missing results in a synthesis (arising from reporting biases).	16 –18
Certainty assessment	15	Describe any methods used to assess certainty (or confidence) in the body of evidence for an outcome.	18

**RESULTS**			
Study selection	16a	Describe the results of the search and selection process, from the number of records identified in the search to the number of studies included in the review, ideally using a flow diagram.	19 – 20<br>Figure 1
	16b	Cite studies that might appear to meet the inclusion criteria, but which were excluded, and explain why they were excluded.	20
Study characteristics	17	Cite each included study and present its characteristics.	Table 3 – 5
Risk of bias in studies	18	Present assessments of risk of bias for each included study.	20-21<br>Table 2
Results of individual studies	19	For all outcomes, present, for each study: (a) summary statistics for each group (where appropriate) and (b) an effect estimate and its precision (e.g. confidence/credible interval), ideally using structured tables or plots.	Table 3 – 5 &<br>Figure 2 – 9
Results of syntheses	20a	For each synthesis, briefly summarise the characteristics and risk of bias among contributing studies.	23 – 34
	20b	Present results of all statistical syntheses conducted. If meta-analysis was done, present for each the summary estimate and its precision (e.g. confidence/credible interval) and measures of statistical heterogeneity. If comparing groups, describe the direction of the effect.	34 – 47
	20c	Present results of all investigations of possible causes of heterogeneity among study results.	34 – 47
	20d	Present results of all sensitivity analyses conducted to assess the robustness of the synthesized results.	34 – 47
Reporting biases	21	Present assessments of risk of bias due to missing results (arising from reporting biases) for each synthesis assessed.	47– 48
Certainty of evidence	22	Present assessments of certainty (or confidence) in the body of evidence for each outcome assessed.	34 – 47
**DISCUSSION**			
Discussion	23a	Provide a general interpretation of the results in the context of other evidence.	57– 62
	23b	Discuss any limitations of the evidence included in the review.	57– 63
	23c	Discuss any limitations of the review processes used.	62– 63
	23d	Discuss implications of the results for practice, policy, and future research.	64 – 65
**OTHER INFORMATION**			
Registration and protocol	24a	Provide registration information for the review, including register name and registration number, or state that the review was not registered.	10
	24b	Indicate where the review protocol can be accessed, or state that a protocol was not prepared.	10
	24c	Describe and explain any amendments to information provided at registration or in the protocol.	Supplementary Table 2
Support	25	Describe sources of financial or non-financial support for the review, and the role of the funders or sponsors in the review.	65
Competing interests	26	Declare any competing interests of review authors.	65
Availability of data, code and other materials	27	Report which of the following are publicly available and where they can be found: template data collection forms; data extracted from included studies; data used for all analyses; analytic code; any other materials used in the review.	65

From: Page MJ, McKenzie JE, Bossuyt PM, Boutron I, Hoffmann TC, Mulrow CD, et al. The PRISMA 2020 statement: an updated guideline for reporting systematic reviews. BMJ 2021;372:n71. doi: 10.1136/bmj.n71 For more information, visit: http://www.prisma-statement.org/

Note. Page numbers referred to the original, unformatted version.

Supplementary Table S2. Summary of Amendments to PROSPERO Protocol (CRD42020148110; aalhamdan et al., 2019)

NO.	Date Published	Description
1	4/12/2023	1. **Updated title** form "A systematic review of the contribution of multisensory processing to memory performance in children (0-12- years)"- to a new title " From Senses to Memory: A Systematic Review and Bayesian Meta-analyses Exploring Multisensory Processing and Working Memory Development."<br><br>2**. Updated Review question** to be " 1. What is the relationship between multisensory processing including (motor reaction times (MRTs) and verbal multisensory tasks), and visual and auditory working memory measures in children from birth to 15 years old? 2-Do audiovisual stimuli contribute more to working memory capacity in comparison to unisensory auditory-alone and/or visual-alone stimuli during childhood?"<br><br>3**. Slight revision to inclusion criteria**- the third inclusion criterion was studies of children aged 0 to 12 years old, however, within the systematic search, it is important to include children from birth to 15 years old, as multisensory facilitation emerges early in life (Lewkowicz & Lickliter, 1994), and reaching maturity late during childhood and early adolescence (Barutchu et al., 2009; Brandwein et al., 2011; Nardini et al., 2008). Meanwhile, we need to update the third point in the exclusion criteria to be "Participants aged over 15 years."<br><br>4. **Updated Participants/population section** to be<br>Inclusion: Children from birth to 15 years old (normal - typically developing).<br>Exclusion: Aged over 15 years old, and visual or auditory impairment.<br><br>5. **Updated the main Outcomes(s) section-** to read- The primary outcome will demonstrate the correlation between measures (1) and (2) above. The secondary outcomes will calculate effect sizes to demonstrate the contribution of multisensory processing (first variable) to memory performance (second variable) in children 0-15 years.<br><br>6. **Updated Data Extraction** to read- The following information from each study was extracted: study details (author(s), year of publication), study purpose (aims as stated in the study), participant information (number of total participants, age [M, (SD), range]), measures of multisensory processing tasks (type of MS task, task

description, and degree of complexity), measures of WM tasks (visual or auditory), and outcomes for both correlation coefficients and effect size analyses.

7. **Updated Strategy for data synthesis** to read - Although the initial intention was to use a narrative approach, upon screening, it was found that there were a satisfactory number of included studies with substantial similarities, enabling the undertaking of meta-analyses. First, a Bayesian meta-analysis of the correlation coefficients will be used to investigate the association between multisensory tasks (including MRTs and verbal multisensory tasks) and working memory. Second, effect sizes will be computed to examine the differences in working memory capacity for audiovisual stimuli vs. auditory stimuli, as well as for audiovisual stimuli vs. visual stimuli (VS). A Bayesian meta-analysis was conducted using the free software JASP; Jeffreys's Amazing Statistics Program 0.16.3.0 (JASP Team, 2022; http://www.jasp-stats.org/), with a random effect model. The prior (estimated) and posterior (observed) odds, 95% credible intervals (95% CI), and sequential analyses will be applied. Robust Bayesian meta-analyses will also be conducted to assess the presence or absence of effect, heterogeneity, and publication bias.

8. **Added information to Analysis of subgroups or subsets section** to read- Results will firstly be categorized according to a type of multisensory tasks (a. Motor reaction times (i.e., measuring how quickly participants press a button), b. Verbal motor multisensory tasks (i.e., assessing accuracy through the number of correct responses as reported verbally and without any time-related data).

9. **Updated type and method of review** to include Bayesian meta-analysis.

Supplementary Table S3. Search Strategy for all Databases

Multisensory	Memory	Children (under 12 years)
**Keywords:**<br>(sound* ADJ2 (vision OR visual OR sight*))<br>(multisensory ADJ2 (integration OR perspective OR processing))<br>(sensory ADJ2 (integration OR cross-modal OR multiple))<br>Intersensory processing<br>((Audiovisual OR audio-visual) ADJ2 (synchrony OR information))<br>Visual auditory integration<br>Audition<br><br>**Subject headings:**<br>intersensory processes/ or sensory integration/<br>visual perception<br>auditory perception	**Keywords:**<br>((working OR short-term OR long-term OR verbal OR visual OR spatial) ADJ2 memory*)<br><br>**Subject headings:**<br>memory/ or long term memory/ or short term memory/ or spatial memory/ or verbal memory/ or visual memory/	**Keywords:**<br>(Child* ADJ2(development OR early OR school age* OR pre-school OR young))<br>((preschool OR pre-school OR school) ADJ2 student*)<br>Birth OR infant OR toddler OR newborn OR child*<br><br>**Subject headings:**<br>elementary school students/ or preschool students/<br>childhood development/ or early childhood development/<br>exp Infant Development/<br><br>Child<br>Child, preschool<br>Infant<br><br>Child development

ADJ – is a proximity operator in PsyINFO(Ovid), MEDLINE (Ovid), and EMBASE (Ovid), that finds words within a certain distance of each other. ADJ2, finds each word within 2 words of the other. This can be represented differently in different databases. It can be used as N2 or NEAR2 OR N/2 in pubMED, Web of science(ISI) and CINAHL(EBSCO).

Supplementary Figures S1- S6. Full Search Strategy for each Databases

Supplementary Figure S1. PsyINFO (Ovid) Database (n = 660)

▼ Search History (27)

# ▲	Searches	Results
1	(sound* adj2 (vision or visual or sight*)).mp. [mp=title, abstract, heading word, table of contents, key concepts, original title, tests & measures, mesh]	745
2	(multisensory adj2 (integration or perspective or processing)).mp. [mp=title, abstract, heading word, table of contents, key concepts, original title, tests & measures, mesh]	1803
3	(sensory adj2 (integration or cross-modal or multiple)).mp. [mp=title, abstract, heading word, table of contents, key concepts, original title, tests & measures, mesh]	5333
4	exp Intersensory Processes/ or Intersensory processing.mp.	6335
5	((Audiovisual or audio-visual) adj2 (synchrony or information)).mp. [mp=title, abstract, heading word, table of contents, key concepts, original title, tests & measures, mesh]	242
6	Visual auditory integration.mp.	20
7	Audition.mp. or exp Auditory Perception/	42089
8	intersensory processes/ or sensory integration/	5426
9	visual perception.mp. or exp Visual Perception/	126348
10	auditory perception.mp. or exp Auditory Perception/	46996
11	9 and 10	7477
12	1 or 2 or 3 or 4 or 5 or 6 or 7 or 8 or 11	52550
13	((working or short-term or long-term or verbal or visual or spatial) adj2 memory*).mp. [mp=title, abstract, heading word, table of contents, key concepts, original title, tests & measures, mesh]	69999
14	memory/ or early memories/ or episodic memory/ or explicit memory/ or implicit memory/ or long term memory/ or prospective memory/ or short term memory/ or spatial memory/ or verbal memory/ or visual memory/ or cued recall/ or free recall/ or "recall (learning)"/	117490
15	13 or 14	142994
16	(Child* adj2 (development or early or school age* or pre-school or young)).mp. [mp=title, abstract, heading word, table of contents, key concepts, original title, tests & measures, mesh]	169666
17	((preschool or pre-school or school) adj2 student*).mp. [mp=title, abstract, heading word, table of contents, key concepts, original title, tests & measures, mesh]	124780
18	(Birth or infant or toddler or newborn or child*).mp. [mp=title, abstract, heading word, table of contents, key concepts, original title, tests & measures, mesh]	833523
19	elementary school students/ or intermediate school students/ or primary school students/	42702
20	early childhood development/ or childhood development/ or infant development/	96436
21	child.mp.	434761
22	preschool students/ or kindergarten students/	15728
23	infant.mp. or exp Infant Development/	80405
24	exp Early Childhood Development/ or exp Childhood Development/ or child development.mp.	117019
25	preschool.mp.	43662
26	16 or 17 or 18 or 19 or 20 or 21 or 22 or 23 or 24 or 25	915138
27	12 and 15 and 26	660

Save Remove Combine with: AND OR

Supplementary Figure S2. MEDLINE (Ovid) Database (*n* = 1031)

ovidsp.dc2.ovid.com.ez.library.latrobe.edu.au/sp-4.02.1a/ovidweb.cgi?&S=IAEBFPDBNPEBBIAIIPCKCFPEFPEKAA00&C=_main&tab=search&Main+Search+Page=1

# ▲	Searches	Results	Type
1	(sound* adj2 (vision or visual or sight*)).mp. [mp=title, abstract, original title, name of substance word, subject heading word, floating sub-heading word, keyword heading word, organism supplementary concept word, protocol supplementary concept word, rare disease supplementary concept word, unique identifier, synonyms]	543	Advanced
2	(multisensory adj2 (integration or perspective or processing)).mp. [mp=title, abstract, original title, name of substance word, subject heading word, floating sub-heading word, keyword heading word, organism supplementary concept word, protocol supplementary concept word, rare disease supplementary concept word, unique identifier, synonyms]	2025	Advanced
3	(sensory adj2 (integration or cross-modal or multiple)).mp. [mp=title, abstract, original title, name of substance word, subject heading word, floating sub-heading word, keyword heading word, organism supplementary concept word, protocol supplementary concept word, rare disease supplementary concept word, unique identifier, synonyms]	3081	Advanced
4	exp Intersensory Processes/ or Intersensory processing.mp.	7	Advanced
5	((Audiovisual or audio-visual) adj2 (synchrony or information)).mp. [mp=title, abstract, original title, name of substance word, subject heading word, floating sub-heading word, keyword heading word, organism supplementary concept word, protocol supplementary concept word, rare disease supplementary concept word, unique identifier, synonyms]	293	Advanced
6	Visual auditory integration.mp.	19	Advanced
7	Audition.mp. or exp Auditory Perception/	74697	Advanced
8	intersensory processes/ or sensory integration/	0	Advanced
9	visual perception.mp. or exp Visual Perception/	219911	Advanced
10	auditory perception.mp. or exp Auditory Perception/	74432	Advanced
11	9 and 10	18210	Advanced
12	1 or 2 or 3 or 4 or 5 or 6 or 7 or 8 or 11	79299	Advanced
13	((working or short-term or long-term or verbal or visual or spatial) adj2 memory*).mp. [mp=title, abstract, original title, name of substance word, subject heading word, floating sub-heading word, keyword heading word, organism supplementary concept word, protocol supplementary concept word, rare disease supplementary concept word, unique identifier, synonyms]	69161	Advanced
14	memory/ or early memories/ or episodic memory/ or explicit memory/ or implicit memory/ or long term memory/ or prospective memory/ or short term memory/ or spatial memory/ or verbal memory/ or visual memory/ or cued recall/ or free recall/ or "recall (learning)"/	91130	Advanced
15	13 or 14	122606	Advanced
16	(Child* adj2 (development or early or school age* or pre-school or young)).mp. [mp=title, abstract, original title, name of substance word, subject heading word, floating sub-heading word, keyword heading word, organism supplementary concept word, protocol supplementary concept word, rare disease supplementary concept word, unique identifier, synonyms]	166269	Advanced
17	((preschool or pre-school or school) adj2 student*).mp. [mp=title, abstract, original title, name of substance word, subject heading word, floating sub-heading word, keyword heading word, organism supplementary concept word, protocol supplementary concept word, rare disease supplementary concept word, unique identifier, synonyms]	18634	Advanced
18	(Birth or infant or toddler or newborn or child*).mp. [mp=title, abstract, original title, name of substance word, subject heading word, floating sub-heading word, keyword heading word, organism supplementary concept word, protocol supplementary concept word, rare disease supplementary concept word, unique identifier, synonyms]	3069477	Advanced
19	elementary school students/ or intermediate school students/ or primary school students/	0	Advanced
20	early childhood development/ or childhood development/ or infant development/	44265	Advanced
21	child.mp.	2032802	Advanced
22	preschool students/ or kindergarten students/	0	Advanced
23	infant.mp. or exp Infant Development/	1191037	Advanced
24	exp Early Childhood Development/ or exp Childhood Development/ or child development.mp.	55092	Advanced
25	preschool.mp.	894023	Advanced
26	16 or 17 or 18 or 19 or 20 or 21 or 22 or 23 or 24 or 25	3082321	Advanced
27	12 and 15 and 26	1031	Advanced

Save Remove Combine with: AND OR

Supplementary Figure S3. EMBASE (Ovid) Database (*n* = 673)

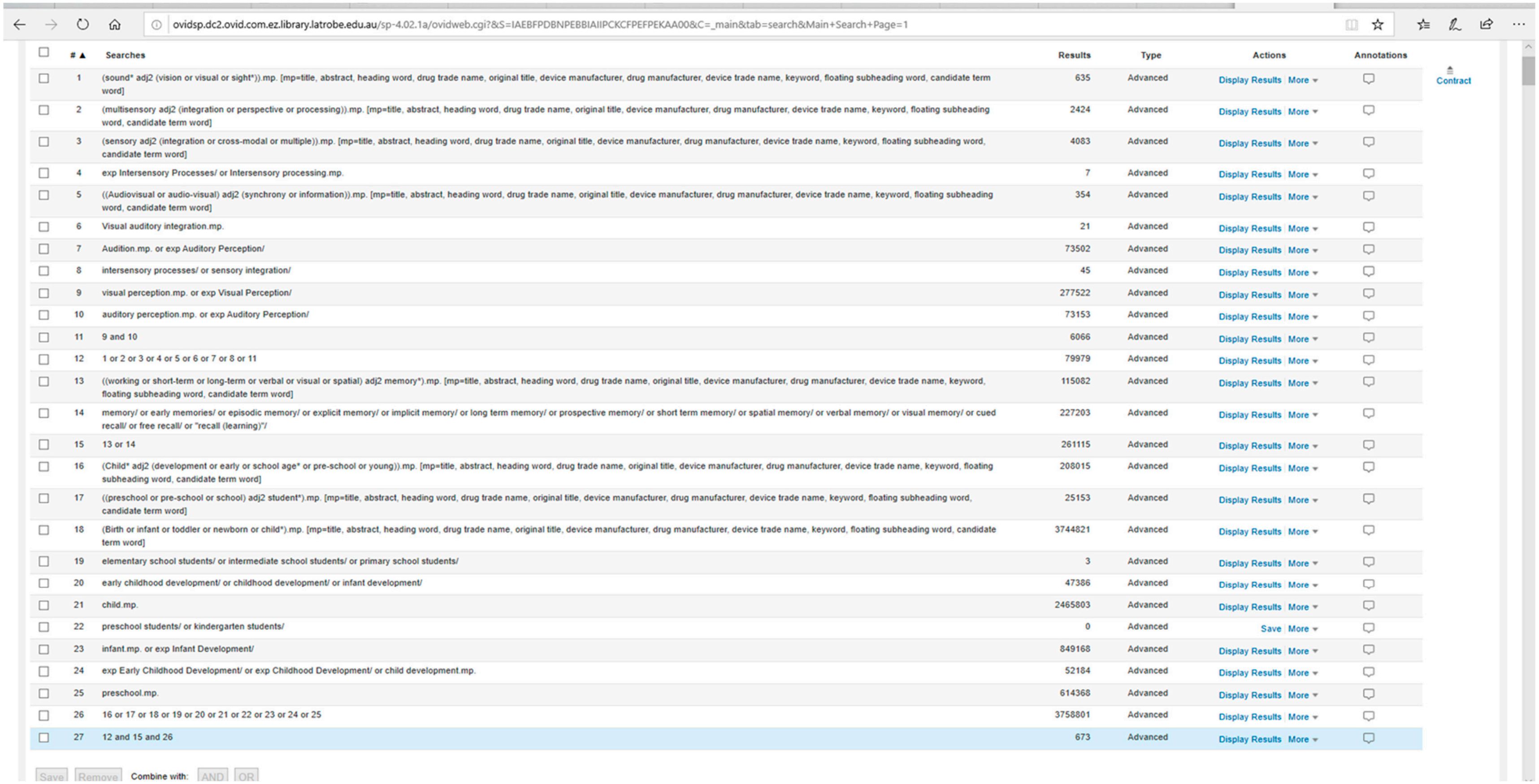

# ▲	Searches	Results	Type	Actions	Annotations
1	(sound* adj2 (vision or visual or sight*)).mp. [mp=title, abstract, heading word, drug trade name, original title, device manufacturer, drug manufacturer, device trade name, keyword, floating subheading word, candidate term word]	635	Advanced	Display Results | More	
2	(multisensory adj2 (integration or perspective or processing)).mp. [mp=title, abstract, heading word, drug trade name, original title, device manufacturer, drug manufacturer, device trade name, keyword, floating subheading word, candidate term word]	2424	Advanced	Display Results | More	
3	(sensory adj2 (integration or cross-modal or multiple)).mp. [mp=title, abstract, heading word, drug trade name, original title, device manufacturer, drug manufacturer, device trade name, keyword, floating subheading word, candidate term word]	4083	Advanced	Display Results | More	
4	exp Intersensory Processes/ or Intersensory processing.mp.	7	Advanced	Display Results | More	
5	((Audiovisual or audio-visual) adj2 (synchrony or information)).mp. [mp=title, abstract, heading word, drug trade name, original title, device manufacturer, drug manufacturer, device trade name, keyword, floating subheading word, candidate term word]	354	Advanced	Display Results | More	
6	Visual auditory integration.mp.	21	Advanced	Display Results | More	
7	Audition.mp. or exp Auditory Perception/	73502	Advanced	Display Results | More	
8	intersensory processes/ or sensory integration/	45	Advanced	Display Results | More	
9	visual perception.mp. or exp Visual Perception/	277522	Advanced	Display Results | More	
10	auditory perception.mp. or exp Auditory Perception/	73153	Advanced	Display Results | More	
11	9 and 10	6066	Advanced	Display Results | More	
12	1 or 2 or 3 or 4 or 5 or 6 or 7 or 8 or 11	79979	Advanced	Display Results | More	
13	((working or short-term or long-term or verbal or visual or spatial) adj2 memory*).mp. [mp=title, abstract, heading word, drug trade name, original title, device manufacturer, drug manufacturer, device trade name, keyword, floating subheading word, candidate term word]	115082	Advanced	Display Results | More	
14	memory/ or early memories/ or episodic memory/ or explicit memory/ or implicit memory/ or long term memory/ or prospective memory/ or short term memory/ or spatial memory/ or verbal memory/ or visual memory/ or cued recall/ or free recall/ or "recall (learning)"/	227203	Advanced	Display Results | More	
15	13 or 14	261115	Advanced	Display Results | More	
16	(Child* adj2 (development or early or school age* or pre-school or young)).mp. [mp=title, abstract, heading word, drug trade name, original title, device manufacturer, drug manufacturer, device trade name, keyword, floating subheading word, candidate term word]	208015	Advanced	Display Results | More	
17	((preschool or pre-school or school) adj2 student*).mp. [mp=title, abstract, heading word, drug trade name, original title, device manufacturer, drug manufacturer, device trade name, keyword, floating subheading word, candidate term word]	25153	Advanced	Display Results | More	
18	(Birth or infant or toddler or newborn or child*).mp. [mp=title, abstract, heading word, drug trade name, original title, device manufacturer, drug manufacturer, device trade name, keyword, floating subheading word, candidate term word]	3744821	Advanced	Display Results | More	
19	elementary school students/ or intermediate school students/ or primary school students/	3	Advanced	Display Results | More	
20	early childhood development/ or childhood development/ or infant development/	47386	Advanced	Display Results | More	
21	child.mp.	2465803	Advanced	Display Results | More	
22	preschool students/ or kindergarten students/	0	Advanced	Save | More	
23	infant.mp. or exp Infant Development/	849168	Advanced	Display Results | More	
24	exp Early Childhood Development/ or exp Childhood Development/ or child development.mp.	52184	Advanced	Display Results | More	
25	preschool.mp.	614368	Advanced	Display Results | More	
26	16 or 17 or 18 or 19 or 20 or 21 or 22 or 23 or 24 or 25	3758801	Advanced	Display Results | More	
27	12 and 15 and 26	673	Advanced	Display Results | More	

Supplementary Figure S4. CINAHL (EBSCO) Database (n = 166)

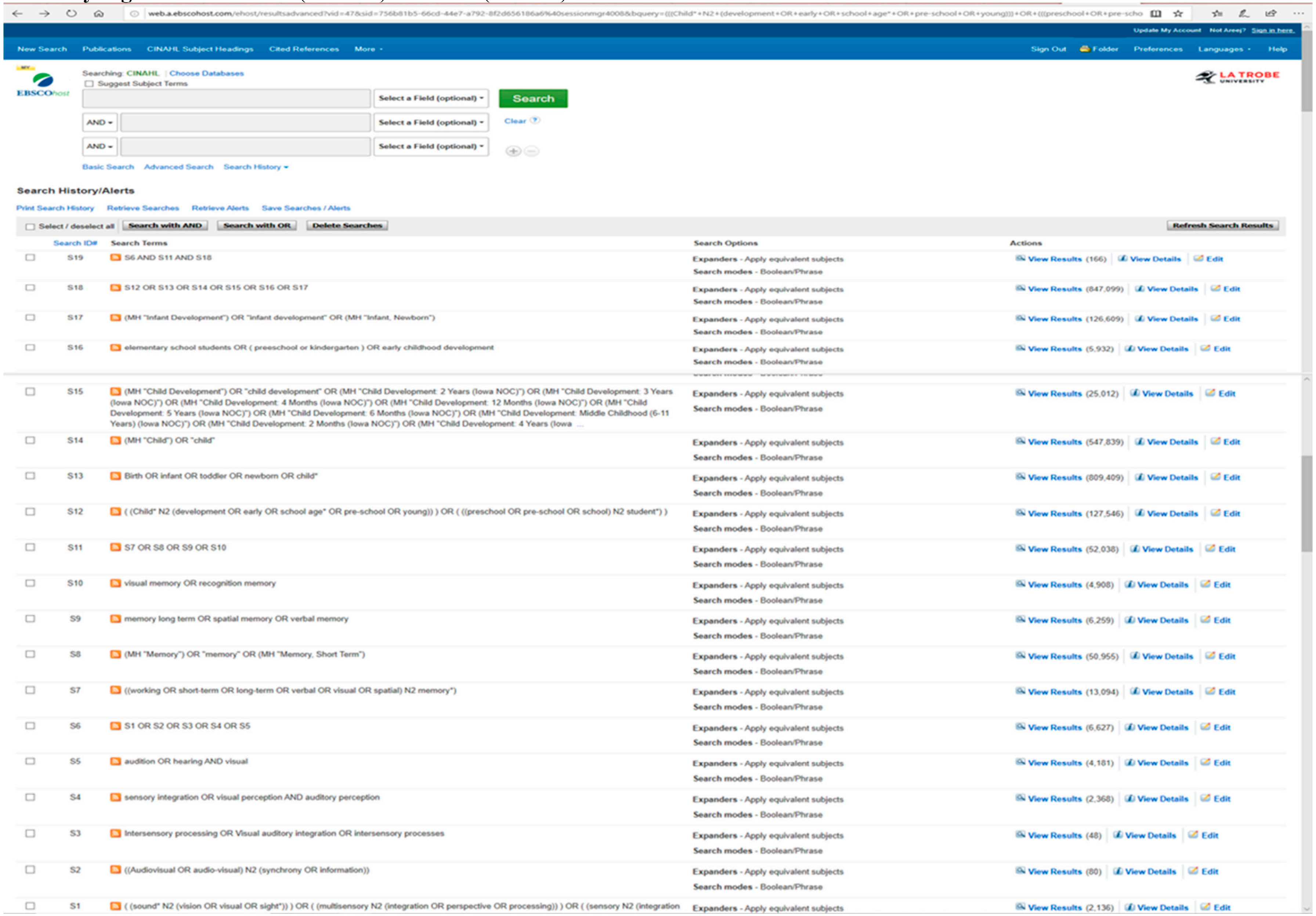

Searching: CINAHL | Choose Databases

Search History/Alerts

Search ID#	Search Terms	Search Options	Actions
S19	S6 AND S11 AND S18	Expanders - Apply equivalent subjects<br>Search modes - Boolean/Phrase	View Results (166) | View Details | Edit
S18	S12 OR S13 OR S14 OR S15 OR S16 OR S17	Expanders - Apply equivalent subjects<br>Search modes - Boolean/Phrase	View Results (847,099) | View Details | Edit
S17	(MH "Infant Development") OR "infant development" OR (MH "Infant, Newborn")	Expanders - Apply equivalent subjects<br>Search modes - Boolean/Phrase	View Results (126,609) | View Details | Edit
S16	elementary school students OR (preeschool or kindergarten) OR early childhood development	Expanders - Apply equivalent subjects<br>Search modes - Boolean/Phrase	View Results (5,932) | View Details | Edit
S15	(MH "Child Development") OR "child development" OR (MH "Child Development: 2 Years (Iowa NOC)") OR (MH "Child Development: 3 Years (Iowa NOC)") OR (MH "Child Development: 4 Months (Iowa NOC)") OR (MH "Child Development: 12 Months (Iowa NOC)") OR (MH "Child Development: 5 Years (Iowa NOC)") OR (MH "Child Development: 6 Months (Iowa NOC)") OR (MH "Child Development: Middle Childhood (6-11 Years) (Iowa NOC)") OR (MH "Child Development: 2 Months (Iowa NOC)") OR (MH "Child Development: 4 Years (Iowa ...	Expanders - Apply equivalent subjects<br>Search modes - Boolean/Phrase	View Results (25,012) | View Details | Edit
S14	(MH "Child") OR "child"	Expanders - Apply equivalent subjects<br>Search modes - Boolean/Phrase	View Results (547,839) | View Details | Edit
S13	Birth OR infant OR toddler OR newborn OR child*	Expanders - Apply equivalent subjects<br>Search modes - Boolean/Phrase	View Results (809,409) | View Details | Edit
S12	((Child* N2 (development OR early OR school age* OR pre-school OR young))) OR (((preschool OR pre-school OR school) N2 student*))	Expanders - Apply equivalent subjects<br>Search modes - Boolean/Phrase	View Results (127,546) | View Details | Edit
S11	S7 OR S8 OR S9 OR S10	Expanders - Apply equivalent subjects<br>Search modes - Boolean/Phrase	View Results (52,038) | View Details | Edit
S10	visual memory OR recognition memory	Expanders - Apply equivalent subjects<br>Search modes - Boolean/Phrase	View Results (4,908) | View Details | Edit
S9	memory long term OR spatial memory OR verbal memory	Expanders - Apply equivalent subjects<br>Search modes - Boolean/Phrase	View Results (6,259) | View Details | Edit
S8	(MH "Memory") OR "memory" OR (MH "Memory, Short Term")	Expanders - Apply equivalent subjects<br>Search modes - Boolean/Phrase	View Results (50,955) | View Details | Edit
S7	((working OR short-term OR long-term OR verbal OR visual OR spatial) N2 memory*)	Expanders - Apply equivalent subjects<br>Search modes - Boolean/Phrase	View Results (13,094) | View Details | Edit
S6	S1 OR S2 OR S3 OR S4 OR S5	Expanders - Apply equivalent subjects<br>Search modes - Boolean/Phrase	View Results (6,627) | View Details | Edit
S5	audition OR hearing AND visual	Expanders - Apply equivalent subjects<br>Search modes - Boolean/Phrase	View Results (4,181) | View Details | Edit
S4	sensory integration OR visual perception AND auditory perception	Expanders - Apply equivalent subjects<br>Search modes - Boolean/Phrase	View Results (2,368) | View Details | Edit
S3	Intersensory processing OR Visual auditory integration OR intersensory processes	Expanders - Apply equivalent subjects<br>Search modes - Boolean/Phrase	View Results (48) | View Details | Edit
S2	((Audiovisual OR audio-visual) N2 (synchrony OR information))	Expanders - Apply equivalent subjects<br>Search modes - Boolean/Phrase	View Results (80) | View Details | Edit
S1	((sound* N2 (vision OR visual OR sight*))) OR ((multisensory N2 (integration OR perspective OR processing))) OR ((sensory N2 (integration	Expanders - Apply equivalent subjects	View Results (2,136) | View Details | Edit

Supplementary Figure S5. PubMed Database (*n* = 1048)

ubmed/advanced

History

Download history Clear history

Search	Add to builder	Query	Items found	Time
#12	Add	Search ((((((((((((sound* N2 (vision OR visual OR sight*)))) OR ((multisensory N2 (integration OR perspective OR processing)))) OR ((sensory N2 (integration OR cross-modal OR multiple)))) OR (((Audiovisual OR audio-visual) N2 (synchrony OR information)))) OR Visual auditory integration) OR Intersensory processing)) OR (((("sensory integration") OR visual perception) AND auditory perception) OR intersensory processes))) AND ((((((working OR short-term OR long-term OR verbal OR visual OR spatial) N2 memory*)))) OR ((((((memory/ OR long term)) OR (memory/ OR short term)) OR (memory/ OR spatial memory)) OR (verbal memory/ OR visual memory/)) OR recognition memory))) AND (((((((((Child* N2(development OR early OR school age* OR pre-school OR young)))) OR (((preschool OR pre-school OR school) N2 student*))) OR (Birth OR infant OR toddler OR newborn OR child*))) OR ((elementary school students OR preschool students*))) OR ((childhood development OR early childhood development OR Infant Development*))) OR ((((child) OR preschool) OR infant) OR child development)) Sort by: Best Match	1048	20:51:24
#11	Add	Search ((((((((Child* N2(development OR early OR school age* OR pre-school OR young)))) OR (((preschool OR pre-school OR school) N2 student*))) OR (Birth OR infant OR toddler OR newborn OR child*))) OR ((elementary school students OR preschool students*))) OR ((childhood development OR early childhood development OR Infant Development*))) OR ((((child) OR preschool) OR infant) OR child development) Sort by: Best Match	3333631	20:50:50
#10	Add	Search (((child) OR preschool) OR infant) OR child development Sort by: Best Match	3017139	20:50:20
#9	Add	Search (childhood development OR early childhood development OR Infant Development*) Sort by: Best Match	384294	20:49:07
#8	Add	Search (elementary school students OR preschool students*) Sort by: Best Match	9251	20:48:19
#7	Add	Search ((((Child* N2(development OR early OR school age* OR pre-school OR young)))) OR (((preschool OR pre-school OR school) N2 student*))) OR (Birth OR infant OR toddler OR newborn OR child*) Sort by: Best Match	3331109	20:47:22
#6	Add	Search (((((working OR short-term OR long-term OR verbal OR visual OR spatial) N2 memory*)))) OR ((((((memory/ OR long term)) OR (memory/ OR short term)) OR (memory/ OR spatial memory)) OR (verbal memory/ OR visual memory/)) OR recognition memory) Sort by: Best Match	1209195	20:46:00
#5	Add	Search (((((memory/ OR long term)) OR (memory/ OR short term)) OR (memory/ OR spatial memory)) OR (verbal memory/ OR visual memory/)) OR recognition memory Sort by: Best Match	1209193	20:44:50
#4	Add	Search (((working OR short-term OR long-term OR verbal OR visual OR spatial) N2 memory*)) Sort by: Best Match	284	20:38:02
#3	Add	Search (((((((((sound* N2 (vision OR visual OR sight*)))) OR ((multisensory N2 (integration OR perspective OR processing)))) OR ((sensory N2 (integration OR cross-modal OR multiple)))) OR (((Audiovisual OR audio-visual) N2 (synchrony OR information)))) OR Visual auditory integration) OR Intersensory processing)) OR (((("sensory integration") OR visual perception) AND auditory perception) OR intersensory processes) Sort by: Best Match	24688	20:36:48
#2	Add	Search ((("sensory integration") OR visual perception) AND auditory perception) OR intersensory processes Sort by: Best Match	23616	20:35:16
#1	Add	Search (((((((sound* N2 (vision OR visual OR sight*)))) OR ((multisensory N2 (integration OR perspective OR processing)))) OR ((sensory N2 (integration OR cross-modal OR multiple)))) OR (((Audiovisual OR audio-visual) N2 (synchrony OR information)))) OR Visual auditory integration) OR Intersensory processing Sort by: Best Match	3164	20:32:39

Supplementary Figure S6. Web of sciences (ISI) Database (*n* = 2430)

apps.webofknowledge.com.ez.library.latrobe.edu.au/UA_AdvancedSearch_input.do?product=UA&search_mode=AdvancedSearch&replaceSetId=&goToPageLoc=SearchHistoryTableBanner&SID=C1smbxHu6RG2CtmqYi3&errorQid=50#Sea

			Combine	Delete
# 15	2,430	#14 AND #9 AND #6<br>*Databases= WOS, CCC, DRCI, KJD, MEDLINE, RSCI, SCIELO Timespan=All years*<br>*Search language=Auto*	☐	☐
# 14	8,995,905	#13 OR #12 OR #11 OR #10<br>*Databases= WOS, CCC, DRCI, KJD, MEDLINE, RSCI, SCIELO Timespan=All years*<br>*Search language=Auto*	☐	☐
# 13	2,329,227	TS=(elementary school students* OR preschool students* OR childhood development* OR early childhood development* OR exp Infant Development* OR preschool* OR Infant* OR Child development)<br>*Databases= WOS, CCC, DRCI, KJD, MEDLINE, RSCI, SCIELO Timespan=All years*<br>*Search language=Auto*	☐	☐
# 12	4,524,465	TS=(Birth* OR infant* OR toddler* OR newborn* OR child)<br>*Databases= WOS, CCC, DRCI, KJD, MEDLINE, RSCI, SCIELO Timespan=All years*<br>*Search language=Auto*	☐	☐
# 11	5,385,205	TS=((Child NEAR/2 development OR early OR school age OR pre-school OR young))<br>*Databases= WOS, CCC, DRCI, KJD, MEDLINE, RSCI, SCIELO Timespan=All years*<br>*Search language=Auto*	☐	☐
# 10	168,758	TS=((preschool OR pre-school OR school) NEAR/2 student*)<br>*Databases= WOS, CCC, DRCI, KJD, MEDLINE, RSCI, SCIELO Timespan=All years*<br>*Search language=Auto*	☐	☐
# 9	875,206	#8 OR #7<br>*Databases= WOS, CCC, DRCI, KJD, MEDLINE, RSCI, SCIELO Timespan=All years*<br>*Search language=Auto*	☐	☐
# 8	875,206	TS=(memory* OR long term memory* OR short term memory* OR spatial memory* OR verbal memory* OR visual memory)<br>*Databases= WOS, CCC, DRCI, KJD, MEDLINE, RSCI, SCIELO Timespan=All years*<br>*Search language=Auto*	☐	☐
# 7	152,570	TS=((working OR short-term OR long-term OR verbal OR visual OR spatial) NEAR/2 memory*)<br>*Databases= WOS, CCC, DRCI, KJD, MEDLINE, RSCI, SCIELO Timespan=All years*<br>*Search language=Auto*	☐	☐
# 6	43,929	#5 OR #4 OR #3 OR #2 OR #1<br>*Databases= WOS, CCC, DRCI, KJD, MEDLINE, RSCI, SCIELO Timespan=All years*<br>*Search language=Auto*	☐	☐
# 5	38,145	TS=(Visual auditory integration* OR Intersensory processing* OR sensory integration* OR intersensory processes* OR visual perception* AND auditory perception)<br>*Databases= WOS, CCC, DRCI, KJD, MEDLINE, RSCI, SCIELO Timespan=All years*<br>*Search language=Auto*	☐	☐
# 4	984	TS=((Audiovisual OR audio-visual) NEAR/2 (synchrony OR information))<br>*Databases= WOS, CCC, DRCI, KJD, MEDLINE, RSCI, SCIELO Timespan=All years*<br>*Search language=Auto*	☐	☐
# 3	6,126	TS=(sensory NEAR/2 (integration OR cross-modal OR multiple))<br>*Databases= WOS, CCC, DRCI, KJD, MEDLINE, RSCI, SCIELO Timespan=All years*<br>*Search language=Auto*	☐	☐
# 2	4,056	TS=(multisensory NEAR/2 (integration OR perspective OR processing))<br>*Databases= WOS, CCC, DRCI, KJD, MEDLINE, RSCI, SCIELO Timespan=All years*<br>*Search language=Auto*	☐	☐
# 1	3,093	TS=(sound* NEAR/2 (vision OR visual OR sight*))<br>*Databases= WOS, CCC, DRCI, KJD, MEDLINE, RSCI, SCIELO Timespan=All years*<br>*Search language=Auto*	☐	☐

Supplementary Table S4. Complete data extractions, including means, N, and SD for each group to calculate effect sizes for group difference studies

	A	B	C	D	E	F	G	H	I	J	K	L	M	N	O
1			DATA ENTRY						RAW DIFFERENCE			STANDARDISED EFFECT SIZE			
2	Citation	Outcome measure	Group 1			Group 2			pooled standard deviation	p-value for difference in SDs	Mean Difference	Effect Size	Standard Error of E.S. estimate	Confidence Interval for Effect Size	
3			mean	n	SD	mean	n	SD						lower	upper
4	Constantinidou et al, 2011 8y	AVS vs.AS	9.45	22	1.84	8.91	22	1.54	1.70	0.21	0.54	0.32	0.30	-0.28	0.91
5	Constantinidou et al, 2011 10y	AVS vs.AS	10.44	18	1.2	9.44	18	1.46	1.34	0.21	1.00	0.75	0.34	0.06	1.41
6	Constantinidou & Evripidou, 2012	AVS vs.AS	11.3	20	1.34	10.95	20	1.63	1.49	0.20	0.35	0.23	0.32	-0.39	0.85
7	Field & Anderson, 1985 cued 5 y	AVS vs.AS	56	80	23	40	80	25	24.02	0.23	16.00	0.67	0.16	0.34	0.98
8	Field & Anderson, 1985 cued 9 y	AVS vs.AS	88	80	14	85	80	12	13.04	0.09	3.00	0.23	0.16	-0.08	0.54
9	Field & Anderson, 1985 free 5 y	AVS vs.AS	77.2	80	90.8	58.8	80	50.1	73.33	0.00	18.40	0.25	0.16	-0.06	0.56
10	Field & Anderson, 1985 free 9 y	AVS vs.AS	413.7	80	194.7	307.5	80	128.3	164.88	0.00	106.20	0.64	0.16	0.32	0.96
11	Gillam et al,1998	AVS vs.AS	75.93	16	24.73	82.18	16	23.93	24.33	0.45	-6.25	-0.26	0.35	-0.95	0.45
12	Gillam et al,1998	AVS vs.AS	77.18	16	25.91	82.5	16	25.07	25.49	0.45	-5.32	-0.21	0.35	-0.90	0.49
13	Hatchette& Evans,1983	AVS vs.AS	13.4	18	2.5	12.3	18	2.30	2.40	0.37	1.10	0.46	0.34	-0.21	1.11
14	Pillai & Yathiraj, 2017b score	AVS vs.AS	60.6	28	9.57	57.53	28	8.3	8.96	0.23	3.07	0.34	0.27	-0.19	0.87
15	Pillai & Yathiraj, 2017b span	AVS vs.AS	3.75	28	0.84	4.1	28	0.73	0.79	0.24	-0.35	-0.44	0.27	-0.97	0.09
16	Constantinidou et al, 2011 8y	AVS vs. VS	9.45	22	1.84	9.32	22	1.64	1.74	0.30	0.13	0.07	0.30	-0.52	0.66
17	Constantinidou et al, 2011 10y	AVS vs. VS	10.44	18	1.2	10.66	18	1.14	1.17	0.42	-0.22	-0.19	0.33	-0.84	0.47
18	Choudhury et al, 2007 "70 ms"	AVS vs. VS	62.6	29	11.20	59.6	29	13.20	12.24	0.19	3.00	0.25	0.26	-0.27	0.76
19	Choudhury et al, 2007"300 ms"	AVS vs. VS	61.12	29	11.33	59.6	29	13.20	12.30	0.21	1.52	0.12	0.26	-0.39	0.64
20	Ortiz-Mantilla et al., 2008	AVS vs. VS	59.2	32	8.50	56.9	32	13.40	11.22	0.01	2.30	0.20	0.25	-0.29	0.69
21	Ortiz-Mantilla et al., 2008	AVS vs. VS	56	32	13.10	57.7	32	15.10	14.14	0.22	-1.70	-0.12	0.25	-0.61	0.37
22	Field & Anderson, 1985 cued 5y	AVS vs. VS	56	80	23	45	80	25	24.02	0.23	11.00	0.46	0.16	0.14	0.77
23	Field & Anderson, 1985 cued 9y	AVS vs. VS	88	80	14	68	80	19	16.69	0.00	20.00	1.20	0.17	0.86	1.53
24	Field & Anderson, 1985 free 5y	AVS vs. VS	77.2	80	90.8	67.6	80	72.5	82.16	0.02	9.60	0.12	0.16	-0.19	0.43
25	Field & Anderson, 1985 free 9 y	AVS vs. VS	413.7	80	194.7	273.4	80	156.6	176.68	0.03	140.30	0.79	0.16	0.47	1.11
26	Gillam et al,1998	AVS vs. VS	75.93	16	24.73	80.31	16	20.92	22.90	0.26	-4.38	-0.19	0.35	-0.88	0.51
27	Gillam et al,1998	AVS vs. VS	77.18	16	25.91	72.81	16	23.46	24.72	0.35	4.37	0.18	0.35	-0.52	0.87
28	Hatchette& Evans,1983 (Vs)	AVS vs. VS	13.4	18	2.5	12.2	18	2.4	2.45	0.43	1.20	0.49	0.34	-0.18	1.14
29	Hatchette& Evans,1983 (Vt)	AVS vs. VS	12.7	18	2.8	13.1	18	2.9	2.85	0.44	-0.40	-0.14	0.33	-0.79	0.51
30	Pillai & Yathiraj, 2017b score	AVS vs. VS	60.6	28	9.57	49.71	28	14.74	12.43	0.01	10.89	0.88	0.28	0.32	1.41
31	Pillai & Yathiraj, 2017b span	AVS vs. VS	3.75	28	0.84	3.1	28	1.82	1.42	0.00	0.65	0.46	0.27	-0.08	0.98
32	Constantinidou et al, 2011 8y	AVS vs. VS	9.45	22	1.84	9.32	22	1.64	1.74	0.30	0.13	0.07	0.30	-0.52	0.66
33	Constantinidou et al, 2011 10y	AVS vs. VS	10.44	18	1.2	10.66	18	1.14	1.17	0.42	-0.22	-0.19	0.33	-0.84	0.47
34	Constantinidou & Evripidou, 2012	AVS vs. VS	11.3	20	1.34	11.65	20	1.13	1.24	0.23	-0.35	-0.28	0.32	-0.90	0.35
35	Note. AVS= Audiovisual Stimuli; AS= Auditory Stimuli; VS= Visual Stimuli														

References

Barutchu, A., Crewther, D. P., & Crewther, S. G. (2009). The race that precedes coactivation: Development of multisensory facilitation in children. Developmental Science, 12(3), 464–473.

Brandwein, A. B., Foxe, J. J., Russo, N. N., Altschuler, T. S., Gomes, H., & Molholm, S. (2011). The development of audiovisual multisensory integration across childhood and early adolescence: A high-density electrical mapping study. Cerebral Cortex, 21(5), 1042–1055.

(Choudhury et al., 2007) Choudhury, N., Leppanen, P. H., Leevers, H. J., & Benasich, A. A. (2007). Infant information processing and family history of specific language impairment: Converging evidence for RAP deficits from two paradigms. *Developmental Science*, *10*(2), 213–236. https://doi.org/10.1111/j.1467-7687.2007.00546.x.

(Constantinidou et al., 2011) Constantinidou, F., Danos, M. A., Nelson, D., & Baker, S. (2011). Effects of modality presentation on working memory in school-age children: Evidence for the pictorial superiority hypothesis. *Child Neuropsychology*, *17*(2), 173–196. https://doi.org/10.1080/09297049.2010.525503.

(Constantinidou & Evripidou, 2012) Constantinidou, F., & Evripidou, C. (2012). Stimulus modality and working memory performance in Greek children with reading disabilities: Additional evidence for the pictorial superiority hypothesis. *Child Neuropsychology*, *18*(3), 256–280. https://doi.org/10.1080/09297049.2011.602013.

(Field & Anderson, 1985) Field, D. E., & Anderson, D. R. (1985). Instruction and modality effects on children's television attention and comprehension. *Journal of Educational Psychology*, *77*(1), 91. https://doi.org/https://psycnet.apa.org/doi/10.1037/0022-0663.77.1.91.

(Gillam et al., 1998) Gillam, R. B., Cowan, N., & Marler, J. A. (1998). Information processing by school-age children with specific language impairment: Evidence from a modality effect paradigm. *Journal of Speech, Language, and Hearing Research*, *41*(4), 913–926. https://doi.org/10.1044/jslhr.4104.913.

(Hatchette & Evans, 1983) Hatchette, R. K., & Evans, J. R. (1983). Auditory-visual and temporal-spatial pattern matching performance of two types of learning-disabled children. *Journal of Learning Disabilities*, *16*(9), 537–541. https://doi.org/10.1177/002221948301600910.

JASP Team. (2022). JASP (Version 0.16.3) [Computer Software]. Available online: https://jasp-stats.org/ (accessed on 30 August 2022).

Lewkowicz, D. J., & Lickliter, R. (1994). The development ofintersensory perception: Comparative perspectives. Psychology Press.

Nardini, M., Jones, P., Bedford, R., & Braddick, O. (2008). Development of cue integration in human navigation. Current biology, 18(9),689–693.

(Ortiz-Mantilla et al., 2008) Ortiz-Mantilla, S., Choudhury, N., Leevers, H., & Benasich, A. A. (2008). Understanding language and cognitive deficits in very low birth weight children. *Developmental Psychobiology: The Journal of the International Society for Developmental Psychobiology*, *50*(2), 107–126. https://doi.org/10.1002/dev.20278.

Page, M. J., McKenzie, J. E., Bossuyt, P. M., Boutron, I., Hoffmann, T. C., Mulrow, C. D., Shamseer, L., Tetzlaff, J. M., & Moher, D. (2021).Updating guidance for reporting systematic reviews: Development of the PRISMA 2020 statement. Journal of Clinical Epidemiology, 134, 103–112.

(Pillai & Yathiraj, 2017b) Pillai, R., & Yathiraj, A. (2017b). Two scoring procedures to evaluate memory and sequencing in auditory, visual and auditory-visual combined modalities. *Hearing, Balance and Communication*, *15*(4), 214–220. https://doi.org/10.1080/21695717.2017.1380966.
